# Leakage-Resistant Multi-Sensor Bearing Fault Diagnosis via Adaptive Time-Frequency Graph Learning and Sensor Reliability-Aware Fusion

**DOI:** 10.3390/s26082484

**Published:** 2026-04-17

**Authors:** Yu Sun, Yihang Qin, Wenhao Chen, Wenhui Zhao, Haoran Sun

**Affiliations:** School of Computer Science and Technology, Changchun University, Changchun 130022, China; suny74@ccu.edu.com (Y.S.); cwhxy2024@163.com (W.C.); 15613381757@163.com (W.Z.); 15044418223@163.com (H.S.)

**Keywords:** rolling axle failure diagnosis, multi-sensor fusion, graph convolutional network, time frequency representation, data leakage protection assessment, sensor reliability weighting, adaptive graph learning, short-time fourier transform

## Abstract

Reliable multi-sensor bearing fault diagnosis is challenged by temporal leakage caused by window-level random splitting, limited modeling of cross-sensor dependencies, and inadequate integration of raw temporal dynamics with time-frequency representations. To address these issues, this study proposes a leakage-resistant multi-sensor diagnosis framework that combines a partition-before-windowing evaluation protocol with adaptive time-frequency graph learning and reliability-aware fusion. Continuous vibration records are first divided into disjoint temporal regions with guard intervals and overlap auditing to suppress time-neighbor leakage. The model then extracts complementary features from a raw-signal branch and a dual-resolution log-STFT branch, while adaptive graph learning captures sample-dependent inter-sensor couplings and sensor reliability weighting highlights informative channels. A cross-gated fusion module further integrates temporal and graph-domain representations in a sample-adaptive manner for final classification. Experiments on a reconstructed nine-class benchmark derived from the HUSTbearing dataset show that the proposed method achieves a Macro-Accuracy of 0.973, a Macro-Recall of 0.964, and a Macro-F1 of 0.954, outperforming representative raw-signal and STFT-based baselines under the same leakage-resistant protocol. These results demonstrate that jointly modeling multi-scale time-frequency structure, dynamic sensor relationships, and reliable evaluation yields an effective and interpretable solution for intelligent bearing fault diagnosis under complex operating conditions.

## 1. Introduction

Bearings are critical components in rotating machinery and are widely used in wind turbines [1], high-speed trains, aero-engines, precision manufacturing equipment, and intelligent production lines [2]. Their health condition directly affects system reliability, operational safety, and maintenance cost. Under long-term exposure to fluctuating loads, impact disturbances, lubrication degradation, and complex operating conditions, bearings are vulnerable to faults such as inner-race, outer-race, and rolling-element damage [3], which may further lead to vibration intensification, thermal instability, efficiency loss, and even catastrophic shutdown. With industrial systems evolving toward high speed, heavy load, continuity, and intelligence [4], vibration-based bearing fault diagnosis has become an important research topic in predictive maintenance and industrial artificial intelligence [5].

Traditional bearing fault diagnosis mainly relies on signal processing and pattern recognition, where discriminative features are manually extracted from the time domain, frequency domain, or envelope spectrum and then classified using shallow models such as support vector machines and random forests [6]. Although such methods are physically interpretable, their performance is often highly dependent on expert knowledge and handcrafted feature design, which limits their robustness under noise, load variation, and complex fault coupling. Recent deep-learning approaches, including CNNs [7], RNNs [8], and their variants, have improved automatic representation learning from vibration data [9]. At the same time, time-frequency analysis methods such as STFT [10] and wavelet transform [11] provide informative representations for non-stationary fault signals. However, many existing studies either focus on raw-signal modeling or treat time-frequency maps as ordinary two-dimensional images [12], making it difficult to jointly capture transient waveform details, cross-scale spectral evolution, and inter-sensor dependencies [13]. In addition, random partitioning after window generation may introduce temporal leakage and lead to overly optimistic evaluation results [14].

Recent studies on rotating-system fault diagnosis have further advanced the field from two complementary perspectives. Spirto et al. enhanced SDP-CNN for gear fault detection under variable working conditions by introducing multi-order tracking filtering, showing that tracking-informed preprocessing can effectively strengthen fault-sensitive representations when rotational speed varies [15]. Chen et al. proposed an interpretable wavelet Kolmogorov–Arnold convolutional LSTM for intelligent fault diagnosis, demonstrating that spatial-temporal feature extraction and model interpretability can be jointly improved within a unified deep architecture [16]. Nevertheless, these recent advances mainly emphasize variable-condition representation enhancement or interpretable spatial-temporal modeling, and do not explicitly address temporal-leakage-resistant evaluation, sample-adaptive sensor-topology learning, and reliability-aware fusion in multi-sensor bearing diagnosis.

In practical monitoring scenarios, fault information is rarely concentrated at a single measurement point [17]. Instead, it is distributed across multiple sensors through structural transmission paths, installation-position differences, and condition-dependent coupling effects [18]. This makes multi-sensor diagnosis inherently a relational learning problem rather than a simple multi-channel classification problem. Graph neural networks offer a natural framework for modeling such dependencies [19], yet many existing methods still rely on fixed or manually defined graph structures that cannot adequately reflect sample-dependent sensor interactions under changing working conditions [20]. Likewise, simple concatenation or static weighting is often insufficient for deeply integrating raw temporal dynamics with graph-domain time-frequency representations [21]. Therefore, a unified framework is still needed to simultaneously address three issues: reliable leakage-resistant evaluation, adaptive modeling of inter-sensor relationships, and effective fusion of raw temporal and time-frequency structural information.

To address these challenges, this paper proposes a leakage-resistant multi-sensor bearing fault diagnosis framework based on adaptive time-frequency graph learning and sensor reliability-aware fusion. Specifically, the study makes three main contributions. First, a strict partition-before-windowing protocol is established to suppress time-neighbor leakage and improve the reliability of experimental evaluation [22]. Second, a dual-branch architecture is designed to jointly exploit raw temporal dynamics and dual-resolution log-STFT representations, while adaptive graph learning and sensor reliability weighting [23] are introduced to model sample-dependent inter-sensor coupling more effectively. Third, a cross-gated fusion mechanism is developed to integrate temporal and graph-domain features in a sample-adaptive manner [24], and its effectiveness is validated on a reconstructed nine-class benchmark derived from the HUSTbearing dataset. Overall, unlike conventional stacked-modular diagnostic pipelines [25], the proposed framework integrates evaluation protocol, representation learning, relation modeling [26], and adaptive fusion into a unified diagnosis paradigm for intelligent bearing fault diagnosis under complex operating conditions [27].

## 2. Related Works

### 2.1. Axial Failure Diagnosis Methods Based on Traditional Signal Processing and Shallow-Level Machine Learning

Early diagnostics of axial failure are mostly based on vibration signal processing and artificial feature construction [28]. Typical methods include envelope analysis, small wave transformation, empirical mode decomposition, frequency spectral analysis, and time-domain/frequency-domain statistical extraction, and then combine with classifiers such as SVM, KNN [29], and random forest to achieve failure identification. The core advantages of such methods lie in their clear physical meaning and strong feature interpretability. They are especially suitable for scenarios with small sample sizes and ample prior knowledge. At the same time, methods such as envelope spectral are more consistent with the periodic impact mechanism caused by axial local defects [30], thus still having high practical value in laboratory scenarios.

But its shortcomings are also very obvious: on the one hand, this type of method highly relies on expert experience, and feature design and parameter setting often lack uniform standards; on the other hand, when conditions change, noise increases, or failure patterns become complex, the stability and generalization ability of artificial features significantly decrease. Therefore, traditional methods are more suitable as failure mechanism analysis and interpretability reference, and are difficult to support Goluban Intelligent Diagnostics in complex industrial scenarios alone.

### 2.2. Deep Learning Methods Based on Raw Time Zone Signals

With the development of deep learning, end-to-end models such as 1D-CNNs, residual networks, and attention-based convolutional architectures have been increasingly applied to raw vibration signals [31]. These methods reduce dependence on manual feature engineering and can automatically learn local impact patterns and nonlinear representations, often achieving higher accuracy on public datasets. Nevertheless, raw-signal models still face limitations in modeling long-range dependencies, multi-scale modulation, and cross-sensor complementarity. Moreover, when the data protocol is not sufficiently strict, sliding-window sampling may lead to highly similar samples across subsets, causing overestimation of generalization performance.

### 2.3. Failure Diagnostic Methods Based on Time Frequency Representation

To better characterize the non-stationary and impact-modulated nature of bearing fault signals, many studies first transform one-dimensional signals into time-frequency representations [32] and then perform classification with 2D-CNNs [33], ResNet variants, or Vision Transformers. Such representations can explicitly reveal localized impacts, band-energy migration, and modulation patterns that are difficult to capture from raw waveforms alone. However, this line of research still has three common limitations: the front-end transformation is often sensitive to manually chosen parameters, the time-frequency conversion and classifier are usually optimized separately rather than jointly, and the two-dimensional representation also increases computational and storage costs.

### 2.4. Troubleshooting Methods Based on Multi-Sensor Information Convergence

Because a single sensor provides only a partial observation of machine condition, multi-sensor fusion has become an important strategy for improving diagnostic robustness. Existing studies have shown that combining multiple vibration channels or heterogeneous sensing modalities can improve fault recognition, especially under low signal-to-noise ratio and complex operating conditions [34]. However, many current multi-sensor approaches still perform fusion at the level of data concatenation, feature concatenation, or decision weighting. As a result, they exploit multi-source complementarity only implicitly, while insufficiently modeling sensor topology, coupling strength, and sample-dependent reliability differences.

### 2.5. Graph Neural Network-Based Axial Carrier Failure Diagnostic Methods

Graph neural networks provide a promising way to represent non-Euclidean relationships among sensors, features, or samples. By introducing graph convolution or graph attention mechanisms, recent studies have shown that GNNs can improve structured modeling of cross-channel dependency and multi-sensor coupling in fault diagnosis tasks. Despite this progress, two limitations remain common. First, many graph structures are still manually defined or constructed offline, which makes it difficult to ensure that the learned topology matches the actual physical interaction pattern among sensors. Second, several graph-based studies still depend on handcrafted features or fixed time-frequency inputs, and therefore have not fully achieved deep end-to-end collaboration from raw-signal representation to adaptive relation learning.

### 2.6. Troubleshooting Methods Based on Migrated Learning and Self-Supervised Learning

Transfer learning, domain adaptation, and self-supervised learning have become important research directions for addressing cross-condition variation, limited labels, and distribution shift in industrial scenarios [35]. These methods can reduce the discrepancy between source and target conditions or improve feature learning with unlabeled data, thereby enhancing generalization under variable speed, variable load, and small-sample settings. However, most of this work focuses primarily on domain shift or label scarcity, while paying relatively less attention to multi-sensor relation modeling, interpretability of fusion mechanisms, and the rigor of leakage-resistant evaluation protocols.

### 2.7. Synthesis of Representative Studies and Positioning of the Present Work

To further clarify the novelty of the proposed framework with respect to the existing literature, Table 1 summarizes the main contributions, representative strengths, and remaining limitations of the major research streams reviewed above. As shown, previous studies have advanced bearing fault diagnosis from handcrafted signal analysis to end-to-end deep learning, time-frequency representation learning, multi-sensor fusion, graph-based relational modeling, and transfer/self-supervised strategies. However, few studies simultaneously address leakage-resistant evaluation, sample-adaptive sensor-topology learning, sensor reliability modeling, and deep fusion between raw temporal dynamics and multi-resolution time-frequency representations. This gap directly motivates the unified framework proposed in this study.

## 3. Methodology

### 3.1. Problem Definitions and Symbol Systems

Setting the original multi-sensor vibration signal from the fth status file, recorded as:

$$S_{f}=[s_{f}(1),s_{f}(2),...,s_{f}(N_{f})]\in R^{N_{f}\times C}$$

Here, $N_{f}$ represents the number of sample points for the file, and C represents the number of sensor channels. According to the code, the default C = 3.

Cutting a sliding window of L length from the original sequence as a training sample:

$$X_{m}=S_{f}[t_{m}:t_{m}+L{]}^{T}\in R^{C\times L},\;y_{m}\in \{1,2,\ldots ,K\}$$

Among them, L = 1024, K = 9, corresponding to nine types of bearing states:

The label set of the reconstructed benchmark is defined as:

Y={BL07,BL14,BL21,IR07,IR14,IR21,OR07,OR14,OR21}

Here, BL, IR, and OR denote ball fault, inner-race fault, and outer-race fault, respectively, whereas the suffixes 07, 14, and 21 denote the three severity levels used in the reconstructed benchmark. These shorthand labels are paper-specific identifiers for the controlled benchmark and are used consistently in the confusion matrix, ROC analysis, and ablation discussion.

### 3.2. Window-Level Data Enhancement and Anti-Shortcut Preprocessing

Independent random scaling is applied to each sensor channel:

$${\tilde{x}}_{c}^{\left(1\right)}=\alpha_{c}x_{c},\;\alpha_{c}∼U(a,b)$$

Defines the current channel standard deviation when Gaussian noise is proportional to the signal standard deviation:

$$\sigma_{c}=Std({\tilde{x}}_{c}^{\left(1\right)})+\varepsilon$$

The noise enhancement process is as follows:

$${\tilde{x}}_{c}^{\left(2\right)}={\tilde{x}}_{c}^{\left(1\right)}+\eta_{c},\;\eta_{c}∼N(0,\sigma_{n}^{2}\sigma_{c}^{2}I)$$

Cycle time offset enhancement uses random sampled offset amounts and cycles of offset:

$${\tilde{x}}_{c}^{\left(3\right)}(t)={\tilde{x}}_{c}^{\left(2\right)}((t-\Delta )\;mod\;L)$$

Randomly select a continuous subrange in the time mask mathcal{I} and place zeros:

$${\tilde{x}}_{c}^{\left(4\right)}(t)=\left\{\begin{array}{ll} 0, & t\in I\\ {\tilde{x}}_{c}^{\left(3\right)}(t), & t\notin I \end{array}\right.$$

Additionally, at random channel decay, a certain sensor decays with probability $p_{d}$:

$$\delta_{c}∼\left\{\begin{array}{ll} U(0,0.2), & with\;probability\;p_{d}\\ 1, & with\;probability\;1-p_{d} \end{array}\right.$$

### 3.3. Dual-Resolution Log-STFT and Shared Spectral Encoder

For each sensor waveform, a short-time Fourier transform (STFT) is computed at two resolutions m:

$$V_{c}^{\left(m\right)}(k,\tau )=\sum_{n=0}^{W_{m}-1}x_{c}[n+\tau H_{m}]\;w_{m}[n]\;\exp\left(-j\frac{2\pi kn}{N_{m}}\right),\;0\leq k\leq \frac{N_{m}}{2},$$

where $N_{m}$, $H_{m}$, and $W_{m}$ denote the FFT size, hop length, and analysis-window length, respectively, and $w_{m}[\cdot ]$ is the analysis window. In this study, the two resolutions use $\left(N_{1},H_{1},W_{1}\right)=\left(128,\;32,\;128\right)\;and\;(N_{2},H_{2},W_{2})=(256,\;64,\;256),$ respectively.

The term “logarithmic STFT” in this paper refers to logarithmic magnitude compression after STFT, rather than a logarithmic frequency axis. Specifically, the compressed spectrogram is defined as:

$$S_{c}^{\left(m\right)}(k,\tau )=\log(1+|V_{c}^{\left(m\right)}(k,\tau )|).$$

Because the two STFT branches have different native resolutions, each spectrogram is interpolated to a common grid of size F×T:

$${\bar{S}}_{c}^{\left(m\right)}=Resize(S_{c}^{\left(m\right)},F,T),\;m\in \{1,\;2\}.$$

The two aligned spectrograms are concatenated along the channel dimension to form the dual-resolution time-frequency tensor of sensor c:

$$Z_{c}=Concat({\bar{S}}_{c}^{\left(1\right)},{\bar{S}}_{c}^{\left(2\right)})\in R^{2\times F\times T}.$$

A shared 2D residual encoder $E_{tf}(\cdot )$ is then applied to each sensor tensor:

$$h_{c}^{\left(0\right)}=E_{tf}(Z_{c})\in R^{d_{0}},\;c=1,\ldots ,C.$$

Stacking the node features of all sensors yields the graph input:

$$H^{\left(0\right)}=[h_{1}^{\left(0\right)};h_{2}^{\left(0\right)};\ldots ;h_{C}^{\left(0\right)}]\in R^{C\times d_{0}}.$$

### 3.4. Adaptive Graph Learning and Graph Propagation

Given the sensor-node matrix $H^{\left(l\right)}\in R^{C\times d_{l}}$ at graph layer l, we first project it into query, key, and value spaces:

$$Q^{\left(l\right)}=H^{\left(l\right)}W_{Q}^{\left(l\right)},\;$$

$$K^{\left(l\right)}=H^{\left(l\right)}W_{K}^{\left(l\right)},\;$$

$$V^{\left(l\right)}=H^{\left(l\right)}W_{V}^{\left(l\right)},$$

where $W_{Q}^{\left(l\right)},W_{K}^{\left(l\right)},and\;W_{V}^{\left(l\right)}$ are trainable projection matrices.

The sample-adaptive pairwise affinity matrix is then computed as:

$$E^{\left(l\right)}=\frac{Q^{\left(l\right)}{K^{\left(l\right)}}^{⊤}}{\sqrt{d_{a}}},$$

where $d_{a}$ is the hidden dimension of the query/key space.

To obtain a directional and row-normalized sensor graph, the adaptive adjacency matrix is defined by row-wise softmax normalization:

$$A^{\left(l\right)}={Softmax}_{row}(E^{\left(l\right)}),$$

such that each row of $A^{\left(l\right)}$ sums to 1. Therefore, $A^{\left(l\right)}(i,j)$ can be interpreted as the amount of information that sensor i assigns to sensor j under the current sample.

If a weak prior graph $A_{0}$ is used in the implementation, the adaptive adjacency can be refined as:

$$A^{\left(l\right)}={Softmax}_{row}(E^{\left(l\right)}+\beta A_{0}),$$

where β is a learnable or fixed trade-off coefficient.

The graph propagation at layer l is written as:

$$H^{\left(l+1\right)}=\phi \left(BN(A^{\left(l\right)}V^{\left(l\right)}W_{O}^{\left(l\right)}+H^{\left(l\right)}W_{R}^{\left(l\right)})\right),$$

where $W_{O}^{\left(l\right)}$ and $W_{R}^{\left(l\right)}$ are trainable parameters, BN(⋅) denotes batch normalization, and ϕ(⋅) denotes the non-linear activation function.

In this study, two adaptive graph blocks are stacked, yielding $H^{\left(1\right)}$ and $H^{\left(2\right)}$ in sequence.

Accordingly, $h_{i}^{\left(2\right)}$ denotes the feature of the i-th sensor node after the second adaptive graph propagation block.

### 3.5. Sensor Reliability Weighting, Graph Readout, and Cross-Gated Fusion

After the second graph block, the graph node matrix is $H^{\left(2\right)}=[h_{1}^{\left(2\right)};h_{2}^{\left(2\right)};\ldots ;h_{C}^{\left(2\right)}]\in R^{C\times d_{2}}.$ To model sample-dependent differences in sensor reliability, a scalar score is assigned to each sensor node:

$$s_{i}={w_{2}}^{⊤}\;\phi (W_{1}h_{i}^{\left(2\right)}+b_{1})+b_{2},\;i=1,\ldots ,C.$$

The normalized sensor reliability weight is then obtained by $\alpha_{i}=\frac{\exp(s_{i})}{\sum_{j=1}^{C}\exp(s_{j})}.$ Using these weights, the weighted graph readout and the mean graph readout are computed as:

$$g_{w}=\sum_{i=1}^{C}\alpha_{i}h_{i}^{\left(2\right)},\;g_{a}=\frac{1}{C}\sum_{i=1}^{C}h_{i}^{\left(2\right)}.$$

The global feature of the graph branch is defined as:

$$g=\phi \left(W_{g}[g_{w};g_{a}]+b_{g}\right).$$

Let r $\in R^{d_{r}}$ denote the feature from the raw time-domain branch and g $\in R^{d_{g}}$ denote the feature from the graph/time-frequency branch. We first project them into a common latent space:

$$\tilde{r}=\phi (W_{r}r+b_{r}),\;\tilde{g}=\phi (W_{t}g+b_{t}),where\;\tilde{r},\tilde{g}\in R^{d_{f}}.$$

The cross-gate vector is computed as:

$$z=\sigma \left(W_{z2}\;\phi \left(W_{z1}\left[\tilde{r};\tilde{g}\right]+b_{z1}\right)+b_{z2}\right),$$

where σ(⋅) is the sigmoid function and z $\in R^{d_{f}}$.

The gate vector is then explicitly used to construct the fused representation:

$$f_{fused}=z⊙\tilde{r}+(1-z)⊙\tilde{g},$$

where ⊙ denotes element-wise multiplication. Therefore, z directly controls the dimension-wise contribution of the raw branch and the graph/time-frequency branch.

To preserve both consistency and discrepancy information between the two branches, the final interaction package is defined as:

$$u=\left[\tilde{r};\tilde{g};f_{fused};\left|\tilde{r}-\tilde{g}\right|;\tilde{r}⊙\tilde{g}\right].$$

The final representation and prediction are obtained by $h_{fuse}=MLP\left(u\right),\hat{y}=Softmax\left(W_{c}h_{fuse}+b_{c}\right).$

### 3.6. Logical Workflow of the Study

**Figure 1:**
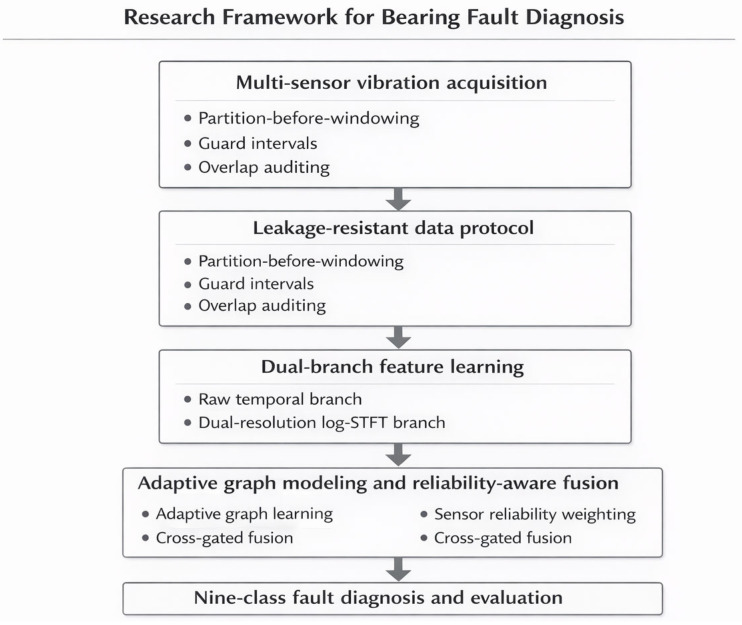
Logical workflow of the present study.

To improve methodological clarity, the complete logic of the present study is summarized in Figure 1. The workflow begins with continuous multi-sensor vibration records from the public HUSTbearing dataset and proceeds through controlled benchmark reformulation, leakage-resistant partition-before-windowing, training-set-only normalization and physically consistent augmentation. The processed samples are then fed into a dual-branch diagnosis model composed of a raw-signal branch and a dual-resolution log-STFT graph branch. In the graph branch, sensor-wise spectral features are encoded, sample-adaptive sensor topology is learned, and sensor reliability is recalibrated before graph-level aggregation. The features from the two branches are finally integrated by a cross-gated fusion module for classification. The experimental stage includes simple same-protocol baselines, repeated-run ablation, and direct leakage verification through overlap auditing and boundary-correlation analysis. This study-level workflow is conceptually different from the internal network architecture, and is therefore illustrated separately to distinguish methodological logic from model implementation details.

## 4. Materials and Methods

### 4.1. Dataset and Signal Pre-Processing

#### 4.1.1. Dataset Description

The experiments in this study are based on the public HUSTbearing dataset released by Zhao, Zio, and Shen [36]. The official HUSTbearing release contains vibration signals from bearings in nine health states under 11 operating conditions, including ten constant-speed conditions (20, 25, 30, 35, 40, 60, 65, 70, 75, and 80 Hz) and one time-varying-speed condition (0–40–0 Hz). The signals were acquired on a Spectra-Quest mechanical fault simulator with an ER-16K bearing and a tri-axial acceleration sensor. The sampling frequency is 25.6 kHz, and each record contains 262,144 sampling points (approximately 10.2 s). In the official release, the second column in the raw files is a redundant speed column and is not used as the ground-truth operating-condition label; the correct operating condition is identified from the filename. These characteristics make HUSTbearing an appropriate benchmark for evaluating leakage-resistant bearing-fault diagnosis under both stationary and non-stationary operating conditions.

It should be emphasized that this study does not directly reuse the original public evaluation setting of HUSTbearing. Instead, we reorganize the continuous recordings into a controlled nine-class closed-set task that is better aligned with the objective of evaluating multi-sensor fusion under a strict anti-leakage protocol. This task-level reformulation improves label clarity and protocol reproducibility; however, it also means that numerical results reported here are not directly comparable to previously published numbers obtained under the native HUSTbearing settings. Therefore, the main comparative claims of this work are made against models re-implemented on the same reconstructed benchmark, using the identical data split and evaluation protocol.

In the reconstructed benchmark used in this study, all 11 operating conditions released with HUSTbearing were retained, namely 20, 25, 30, 35, 40, 60, 65, 70, 75, 80, and 0–40–0 Hz. Following the official HUSTbearing convention, the operating condition of each record was identified from the filename, whereas the second column in the raw file was treated as a redundant speed column rather than as an independent diagnostic variable. Consequently, the present benchmark is condition-overlapping but time-disjoint: the operating conditions are retained as within-class distributional variation under the same health-state label, whereas the training, validation, and test sets are separated along the temporal axis. Category definitions of the controlled nine-class benchmark are listed in Table 2.

#### 4.1.2. Signal Pre-Processing and Sample Construction

To construct diagnostically meaningful and leakage-resistant samples, each raw continuous record was first numerically cleaned by removing invalid entries and retaining only synchronous multi-sensor sequences. The cleaned long sequence was then partitioned before window generation, rather than after window generation, in order to avoid the well-known time-neighbor leakage problem in bearing-fault diagnosis [37]. Specifically, macro-block partitioning was used whenever possible: each continuous record was first divided into macro-blocks of length 8192, which were then assigned to the training, validation, and test sets at a ratio of 70%/15%/15%. Guard intervals were inserted between adjacent subsets. If an individual record was not sufficiently long to support the macro-block strategy, the split fell back to continuous interval partitioning with an explicit guard zone. Only after subset assignment were windows of length 1024 generated independently within each subset. In this way, temporally adjacent segments were prevented from entering different subsets, thereby reducing pseudo-generalization caused by highly similar neighboring windows.

After splitting, two post-split audits were further conducted for each subset pair (train–validation, train–test, and validation–test) [38]. The exact-overlap audit compared the tuple {record ID, start index, end index, sensor group, and class label} and required zero identical tuples across subsets. The near-duplicate audit was performed on standardized and downsampled windows using quantized binary hash signatures, and any flagged candidates were traced back to their original temporal indices and removed if necessary. These procedures provided an additional safeguard against hidden duplication and temporal shortcut learning.

Signal pre-processing followed the principle of training-set-only statistical normalization under physically meaningful constraints. More specifically, all normalization statistics were estimated using the training set only, after which each window underwent four operations in sequence: mean removal, first-order differencing, per-channel z-score normalization, and amplitude clipping. Mean removal suppresses sensor offset and low-frequency baseline drift; first-order differencing enhances local transients induced by impact-type faults; per-channel normalization reduces scale discrepancy across windows and sensors; and amplitude clipping limits the destabilizing effect of occasional extreme peaks. This pre-processing pipeline does not aim to over-whiten the vibration signal, but rather to stabilize its numerical distribution while preserving fault-related mechanical structure for downstream learning.

To further enhance robustness, physically consistent augmentation was applied only during training, including random amplitude scaling, Gaussian noise perturbation proportional to the signal standard deviation, limited cyclic time shifting [39], local time masking, and random channel attenuation. In addition, Mixup was adopted to alleviate overfitting to overly sharp class boundaries. In summary, the pre-processing stage in this study can be summarized as follows: training-set-only normalization was used to prevent statistical leakage; window-level standardization was used to preserve fault-sensitive transients while stabilizing the input distribution; and physically plausible augmentation was used to improve robustness against measurement noise, installation variation, local information loss, and sensor unreliability.

### 4.2. Experimental Environment Setup

Table 3 reports the hardware and software environment used in this study. To improve reproducibility and to make the implementation of the proposed model fully transparent, Table 4 summarizes all core hyperparameters and their values, including data construction, preprocessing, spectral transformation, graph learning, fusion, optimization, and statistical evaluation settings.

To make the computational discussion deployment-relevant rather than hardware-agnostic, we additionally profiled the full diagnostic pipeline under the software/hardware environment listed in Table 3. For each model, we report the number of trainable parameters, peak GPU memory during training, mean wall-clock time per training epoch, total training time to the selected checkpoint, batch-1 inference latency, and batch-64 throughput. Inference timing was measured for the complete forward pipeline from a normalized three-sensor window to class logits, including dual-resolution STFT generation and graph reasoning, after 100 warm-up iterations and over 500 timed iterations with model.eval() and torch.no_grad(); disk I/O was excluded. Because the sampling frequency is 25.6 kHz and the input window length is 1024 points, one analysis window corresponds to approximately 40 ms of signal. We therefore use 40 ms only as a task-specific reference budget for per-window online diagnosis on the present benchmark, while avoiding any hardware-independent claim of universal real-time feasibility.

### 4.3. Experimental Settings

Detailed descriptions of the reconstructed benchmark, leakage-resistant splitting strategy, and signal pre-processing pipeline have been provided in Section 4.1. Here, we focus on model training, optimization, and evaluation settings. The public materials show that HUSTbearing consists of a combination of 9 health states and 11 operating conditions, with a total of 99 sets of continuous vibration records; the experimental platform was the Spectra-Quest mechanical failure simulation laboratory, with the tested bearing model ER-16K, sampling frequency of 25.6 kHz, and each record length of 262,144 points, corresponding to approximately 10.2 s continuous vibration signal. Unlike experiments aimed directly at cross-condition migration, this paper defines “health status” as a supervisory category, retaining different conditions as sources of distributed change within the same class, rather than explicitly introducing domain adaptation or domain generalization training mechanisms. Such a design allows the experimental focus to be focused on whether the proposed model can learn stable and physically meaningful fault determination characterizations in the context of real-world condition perturbations by relying on multi-sensor time-zone-frequency-graph structure joint modeling. In other words, the experimental goal of this paper is not to pursue surface optimization after overlaying multiple training techniques under complex settings, but to validate the structural validity and statistical reliability of the proposed method itself on a clearly defined, protocol-strict, and clearly bounded nine-class identification benchmark. The public note also points out that the second column in the original file is the redundant speed column, which has no independent diagnostic significance in constant-rate data, and that the actual condition should be based on file name identification. Therefore, this paper follows this convention in the data curation phase, treating condition information as a metadata management item, rather than as an alternative source for fault category labels.

The present benchmark is not designed as a leave-one-condition-out, domain-adaptation, or cross-condition generalization setting. Instead, the operating conditions are retained as within-class distributional variation under the same health-state label. Accordingly, the training, validation, and test sets are generated by temporal partitioning within continuous records using the partition-before-windowing protocol, rather than by holding out entire operating conditions. Consequently, the three subsets may all contain samples originating from the same set of operating conditions, but from different non-overlapping time regions. Therefore, the benchmark evaluates leakage-resistant temporal generalization under condition diversity, rather than explicit cross-condition transfer.

In terms of sample construction and data partitioning, this paper follows the basic principle of “partitioning continuous signals first, then cutting windows within each partition” to avoid the time-neighborhood leakage problem that is extremely common in axle failure diagnosis studies. Specifically, the original long sequence is first cleaned and converted into a computable synchronous vibration channel matrix, and then a sample is constructed with a fixed window of length of 1024. This scale is chosen because it can retain diagnostic-value structural information such as failure shock, periodic modulation, and local band energy redistribution while maintaining sufficient time resolution. To suppress pseudo-generalization caused by adjacent windows, this paper prioritizes a macro-blocking strategy: first partitioning macro time blocks of length 8192 on each continuous record, then allocating them to the training, validation, and test sets at a ratio of 70%/15%/15%, while setting up protection blocks between adjacent subsets; if individual records cannot reliably meet the macro-blocking protocol, then regressing to continuous interval partitioning, and explicitly retaining protection intervals of 4096 sampling points. After the zoning is completed, the training, validation, and test windows are generated independently within their respective regions at 1024 steps each. The core goal of this process is not to “artificially reduce the number of samples,” but rather to avoid simultaneously assigning nearly contiguous or even highly duplicated time segments to different subsets, thereby ensuring that test performance truly reflects the model‘s ability to discern unseen time segments, rather than its ability to remember homologous signals.

In terms of input standardization and training configuration, this paper attempts to achieve as much balance as possible between physical consistency and statistical stability. All training statistics are estimated by the training set only, and then de-mean, first-order variance, channel z-score standardization, and width trimming are performed sequentially for each window to suppress sensor zero drift, local DC bias, and anomalous spikes from network training, while enhancing transient response to impact-type failures. To improve the robustness of the model under class-specific condition changes, installation differences, and measurement noise, the training phase introduces enhancements such as random amplitude scaling, Gaussian noise perturbation, finite time offset, local time masking, and random channel decay, and combines label smoothing with Mixup regularization to reduce the model‘s overdependence on a particular local amplitude pattern or on the accidental response of a single sensor. The model training uses the AdamW optimizer, with an initial learning rate set to 1 × 10^−3^, weighted decay to 1 × 10^−4^, and a total of 200 training rounds; the learning rate scheduling uses a strategy of 5 warm-up rounds combined with cosine damping, with a minimum learning rate of 1 × 10^−6^. To improve optimized stability under category imbalances, the loss function adopts soft target focus cross-entropy with category weights, where the label smoothing coefficient is set to 0.05 and the focus coefficient γ is set to 1.0. During training, a gradient clipping with a maximum norm of 3.0 is used and an exponential Moving-average model with a decay coefficient of 0.999 is introduced to validate against optimal model preservation to reduce the impact of later parameter jitter on generalized performance evaluation.

In the construction of time-frequency branches, this paper adopts a dual-resolution Short-time Fourier transform as an explicit frequency-domain a priori for the network. Specifically, the two sets of STFT parameters are set to (n_fft, hop, win) = (128, 32, 128) and (256, 64, 256), respectively, thereby forming a complementary representation between the thinner time resolution and the higher frequency resolution, and input spectral encoders after interpolation alignment to the unified time-frequency grid. The original time zone branch, dual-resolution time frequency branch, and subsequent adaptive graph convolutional modules jointly constitute the core characterization path of this paper: the former is responsible for extracting impact and waveform evolution patterns, the latter is responsible for mining spectral energy migration, multi-scale modulation, and local band anomalies, while the graph structure learning module further characterizes coupling relationships and reliability differences between multiple sensors. As the focus of this paper is to verify the validity of the fusion framework itself, the model chooses to verify the set macro mean F1 value as the main criterion, triggering an early stop if this metric no longer improves within 20 consecutive epochs during training; finally reporting results such as accuracy, macro mean F1, confusion matrix, ROC curve, PR curve, and calibration error on an independent test set. For high-level diagnostic studies, a single accuracy rate is often insufficient to fully characterize model excellence and inferiority. Therefore, this paper simultaneously reports category-level accuracy, recall rate, and F1 values, and evaluates the reliability of output probabilities through reliability graphs and expected calibration errors, thereby making the conclusion not only stop at “scoring correctly”, but further extend to “scoring reliably, scoring reliably”.

In addition, considering that mechanical fault diagnosis models are extremely prone to being misled by pseudo-correlated patterns in practice, this paper also designs additional robustness validation. Specifically, time disorder transformations and symbol inversion transformations can be further introduced in the testing phase to examine the performance changes of the model under non-physical destruction and symbol perturbation. If the model mainly relies on real chronological structures, shock cycles, and sensor coupling relationships, then it should experience significant performance degradation under these disruptive transformations; conversely, if the model still maintains unusually high performance under such non-physical transformations, this often means that it has learned shortcut characteristics unrelated to the mechanism of failure. Through this supplementary experiment, this paper examines not only whether the model is effective, but also why it is effective, thereby further enhancing the explanatory strength and persuasiveness of the entire experimental system.

To avoid over-interpreting single-run fluctuations in the ablation study, all ablation variants were additionally evaluated over *n* = 10 independent runs with different random seeds while keeping the reconstructed benchmark, train/validation/test split, preprocessing pipeline, augmentation policy, and model-specific hyperparameter settings fixed. The sources of stochasticity included parameter initialization, mini-batch order, Mixup pairing, and random augmentation operations. The primary inferential endpoint was test Macro-F1, while Macro-Accuracy and Macro-Recall were reported as secondary descriptive metrics. For each ablation variant, results are reported as mean ± standard deviation across runs. Statistical significance was assessed by two-sided Wilcoxon signed-rank tests on per-run Macro-F1 values against the full model, and Holm–Bonferroni correction was applied to control the family-wise error rate across multiple ablation comparisons. Effect sizes were additionally reported as rank-biserial correlations.

To explicitly assess residual temporal dependence across subset boundaries, we introduced a blockwise boundary-correlation analysis. For each continuous record and each sensor channel, let x denote the terminal macro-block of the upstream subset and y denote the initial macro-block of the downstream subset after exclusion of the guard zone. We computed the normalized cross-correlation $\rho_{x,y}(\tau )=corr(x_{t},y_{t+\tau }),\tau =0,1,\ldots ,\tau_{\max}$, where $\tau_{\max}$ was set to $\left[\tau_{m}ax\right]$. For each boundary pair, we summarized both the lag-0 correlation $\rho_{x,y}(0)$ and the maximum absolute cross-correlation $\underset{\tau }{\max}|\rho_{x,y}(\tau )|$. These quantities were then aggregated across records and channels and compared with the corresponding within-region adjacent-block correlation as a reference for ordinary short-range temporal dependence. A low cross-boundary correlation after guard-zone enforcement indicates that the final split is not only index-disjoint by construction, but also empirically decorrelated at the block level.

### 4.4. Comparable Baselines on the Reconstructed Benchmark

Because the benchmark used in this study is reconstructed from HUSTbearing at the task level rather than directly inherited from the original public evaluation protocol, same-protocol reference baselines are necessary for a fair and verifiable assessment. In addition to the ablation variants derived from our own framework, we therefore implemented a set of simple external baselines on the identical benchmark.

The first baseline, Raw-1D CNN, directly takes the three-channel vibration window as input and applies a standard stack of one-dimensional convolution, batch normalization, nonlinear activation, max-pooling, and global average pooling layers, followed by a fully connected classifier. The second baseline, Raw-1D ResNet18, adapts a standard ResNet-style backbone to multichannel one-dimensional vibration signals.

The third baseline, STFT+2D CNN, converts each window into a single-resolution logarithmic STFT representation and stacks the three sensor spectrograms as a three-channel image for classification by a standard two-dimensional CNN. The fourth baseline, STFT+ResNet18, uses the same STFT representation but replaces the 2D CNN backbone with a standard ResNet18 encoder.

To ensure fairness, all baselines are trained and evaluated under exactly the same protocol as the proposed method: the same nine-class benchmark, the same leakage-resistant train/validation/test split, the same window length, the same guard-interval rule, the same training-set-only normalization, the same early-stopping criterion, and the same evaluation metrics. When augmentation is enabled, it is applied in the signal domain before STFT generation so that both raw-signal and STFT-based models are exposed to the same augmented data distribution. Consequently, the comparison isolates the contribution of model design rather than differences in data partitioning or optimization budget.

In addition to diagnostic accuracy, all baseline models were also compared under an identical computational profiling protocol. This comparison was performed because training epochs alone do not provide a deployment-relevant measure of efficiency. Accordingly, each method was evaluated using the same hardware, preprocessing pipeline, input length, and timing procedure, and was compared in terms of trainable parameters, peak GPU memory, mean training time per epoch, total training time to the selected checkpoint, batch-1 latency, and batch-64 throughput.

Beyond the same-protocol re-implementation baselines described above, we further conducted a literature-level contextual comparison using representative HUSTbearing-based studies reported under their original experimental settings. This additional comparison is included because HUSTbearing is a widely used public benchmark and benchmark-level positioning is informative for readers. However, published HUSTbearing studies differ substantially in class organization, operating-condition protocol, sensor configuration, sample length, preprocessing, and, critically, data-splitting strategy. Therefore, the resulting numerical values are not interpreted here as strictly head-to-head evidence of superiority. Instead, the cross-study comparison is used to position the proposed method with respect to four complementary dimensions: diagnostic performance, methodological family, robustness to noise or condition variation, and reported computational burden. Accordingly, the direct fairness claim of this paper remains anchored to the same reconstructed benchmark and leakage-resistant protocol, whereas the literature comparison serves as broader benchmark-level contextualization.

### 4.5. Model Overall Framework Explanation

The model proposed in this paper is essentially a leak-suppressive dual-branch graph fusion framework for multi-sensor bearing failure diagnosis. It does not simply mechanically stack together time-domain characteristics, frequency-domain characteristics, and graph convolutional modules, but incorporates raw vibration responses, multi-resolution time-frequency representations, and multi-sensor coupling relationships within the same discriminatory system under strict data protocol constraints. Its core idea can be summarized as follows: first, through strict sample construction and leak-proof division, it ensures that the model learns stable fault mechanism information, rather than time proximity redundancy between adjacent windows; then, it extracts complementary characterization in the original time zone and multi-resolution time zone, respectively; and finally, through adaptive map learning and cross-branch gate control fusion, it establishes a unified discriminative expression between “local impact information”, “spectral structure information” and “sensor coupling information”, thereby completing nine high-reliability fault identification.

Starting from the input layer, the model receives a three-channel synchronous vibration window X ∈ R3 × 1024. Before entering the deep network, the signal is first normalized at the window level, including demianization, first-order differential, channel z-score normalization, and width trimming. This step is not a numerical cleanup in the usual sense, but rather to isolate slow-change drift, DC bias, and occasional extreme peaks in mechanical systems from fault-sensitive information, allowing subsequent networks to focus more on truly diagnostic structures such as shock, modulation, and band energy migration. At the same time, enhancement strategies such as amplitude scaling, noise perturbation, time offset, local masking, and channel decay are introduced in the training phase to simulate sample fluctuations, measure noise, and sensor instability that are common in real-world monitoring environments, thereby improving the model‘s robustness to complex condition perturbations. More importantly, the entire set of input pipelines is constrained by a “partitioning first, cutting windows later” leak-proof protocol, that is, first implementing macro-blocking and protective interval control on the original continuous signal, then generating window samples within their respective subsets, so the test results can more truthfully reflect the model‘s ability to generalize to unseen time segments, rather than the memory ability of adjacent segments.

At the level of characterization learning, the model adopts a two-branch parallel structure. The upper branch is the original time zone branch, whose main task is to extract information such as local shocks, phase evolution, periodic repetition, and time sequence microstructures from vibration waveforms that do not undergo explicit spectrum transformation. This branch consists of a one-dimensional convolutional stem and multi-layer residual 1D modules that gradually expand the receptive field while maintaining sensitivity to short-term shock characteristics, ultimately obtaining a compact time zone characterization fraw through pooling and linear projection. The significance of this branch is to preserve the characterization form that most closely approximates the behavior of mechanical dynamics in the original signal, because many fault signals do not initially manifest as apparent band anomalies, but are first embodied in transient shock sequences, waveform peaks, and local time structures. Therefore, if one relies only on spectrum branches, it is easy to lose some early signs of failure that have not yet fully spread to the frequency domain; and the original time domain branches precisely compensate for this shortcoming.

The next branch is the Adaptive Time Frequency Graph branch, which is also where the key innovation of the entire framework lies. This branch first performs a dual-resolution logarithmic STFT on the input signal, using two sets of parameters with higher time resolution and higher frequency resolution, respectively, to uniformly map the failure modulation structure at different scales to the same time frequency representation space. Next, a two-dimensional convolutional encoder is applied to each sensor separately to extract features such as local spectral texture, harmonic structure, and band energy redistribution, and compress the corresponding time frequency representation of each sensor into one node feature in the graph. This way, the multi-sensor system is no longer just a “multi-column input,” but is formally promoted to a graph structure object with node semantics. Next, the model learns the dynamic contiguity between sensors through two layers of AdaptiveGraphBlocks. The graph here is not a predefined fixed topology, but is adaptively generated by node characteristics and fused with a prior sensor graph, achieving a balance between data-driven and structurally prior. The resulting proximity matrix is no longer just a mathematical weight of attention, but can be interpreted as the coupling strength, information transmission direction, and interdependence of different sensors on the same failure state. Furthermore, SensorReliabilityGate assigns soft weights to individual sensors, achieving dynamic recalibration of multi-sensor contributions, enabling the model to maintain stable judgment through the remaining channels even when individual sensors are noise-contaminated or fail locally. Finally, this branch output graph represents an fgraph, which condenses the synergy and reliability structure between multi-sensor time-frequency responses.

For reproducibility, the exact mathematical definitions of the dual-resolution log-STFT, the adaptive adjacency matrix $A^{\left(l\right)}$, the layer-wise node states $h_{i}^{\left(l\right)}$, the sensor reliability weights $\alpha_{i}$, and the cross-gate vector z are given in Section 3.3, Section 3.4 and Section 3.5. In particular, the gate vector is not an auxiliary statistic; it is explicitly used to form $f_{fused}=z⊙\tilde{r}+(1-z)⊙\tilde{g}$.

At the decision level, the model does not simply join fraw and fgraph directly, but introduces the Cross-Gated Fusion module for cross-branch gate-controlled fusion. The essence of this step is to let the model automatically decide whether to trust raw time zone information more, or trust graph structure-driven time frequency coupling information more, under different samples, different failure states. Specifically, the model first projects the two branches into a unified hidden space, then generates dimensional fusion weights through the gate function, thereby constructing the fusion representation ffused. The reason this design is superior to ordinary coupling is that although raw time zone branches and graph time frequency branches are complementary, their discriminatory advantages on different failure types are not constant. For example, for more impactful, time-domain-significant failures, the model may rely more on fraw; while for more complex spectrum-structured failures with more significant cross-sensor relationships, the model may rely more on fgraph. Cross-Gated Fusion transforms this “branch reliability assignment” from an artificial empirical judgment to a learnable adaptive mechanism, so it is not a simple fusion module, but a key element in the entire framework for implementing sample-level discriminative adaptation.

In the final classification phase, the model combines fraw, fgraph, and diffused with their differentials ∣fraw − fgraph∣ to form a feature package, which is then input to the Multilayer perceptron classification header to output nine types of fault logits. The addition of differentials here is not a formal supplementation, but rather to explicitly preserve inconsistent information between the two branches. Because in complex failure scenarios, “two branches giving different judgment grounds” is itself a high-value discriminatory signal, which often corresponds to structural differences between time domain impact and time frequency coupling, and these differences may precisely be the key to distinguishing similar failure categories. At the same time, the combination of soft target focal cross-entropy with category weights, label smoothing, Mixup, AdamW, warm-up and cosine cooling, gradient trimming, EMA, and early stop strategies in the training process together ensure the stability and generalization of the model at the optimization level. Therefore, the entire framework is not “a complex network,” but a set of systematic diagnostic schemes ranging from data protocols, feature extraction, relational modeling, branch fusion, to robust optimization with closed loops.

From an academic point of view, the real contribution of this model framework is not just to improve the accuracy by a few percentage points, but to answer several key questions in multi-sensor axle failure diagnosis at the structural level: first, how to construct reliable deep diagnostic experiments under strict leak prevention premise; second, how to unify raw time zone information and multi-resolution time frequency information into a complementary characterization system; third, how to elevate the coupling relationship between multi-sensors from “input channel superposition” to “learning graph structure”; and fourth, how to achieve branch-level adaptive reliability allocations under different samples, different failure modes.

**Figure 2:**
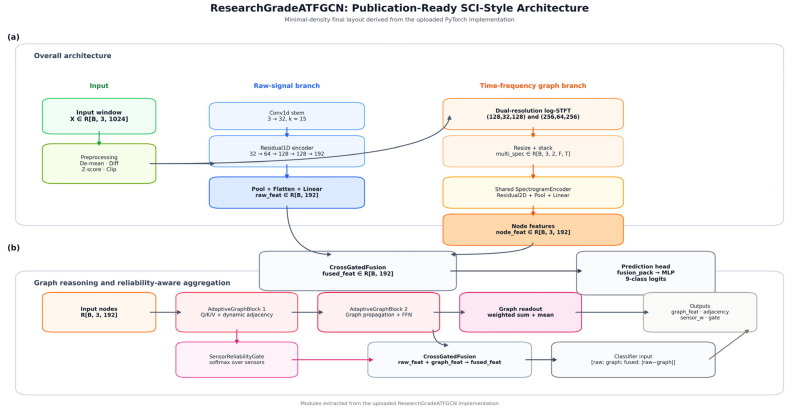
This Institute presents model architecture. (**a**) Original Signal Branch; (**b**) Video Graph Branch.

**Figure 3:**
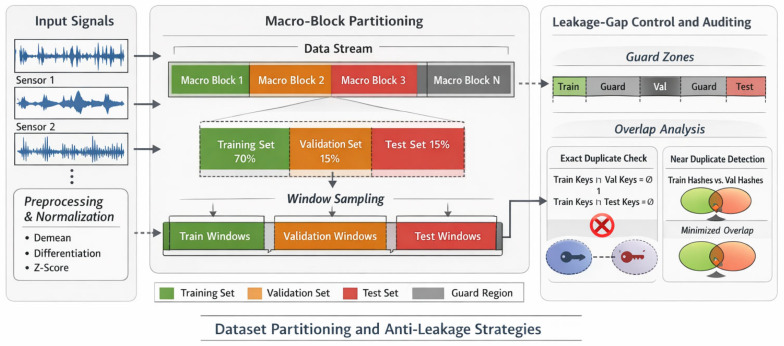
Leakage-prevention data partitioning strategy.

To make the internal design of the proposed network directly interpretable, Figure 2 presents a module-level architecture of the model. Specifically, the figure explicitly visualizes the input representation, the raw-signal branch, the dual-resolution log-STFT graph branch, adaptive graph learning, sensor reliability weighting, the cross-gated fusion module, and the final classification head. Figure 3, by contrast, is reserved for the leakage-prevention mechanism used in benchmark construction and is therefore conceptually different from the internal network architecture.

## 5. Results

### 5.1. Empirical Verification of Leakage Prevention

Because leakage control is a central methodological claim of this study, we first report direct empirical verification of the final partition. Table 5 summarizes the post-split audit results for all subset pairs. The exact-overlap audit yielded 0, 0, and 0 duplicate windows for the train–validation, train–test, and validation–test pairs, respectively. The near-duplicate audit initially flagged 41, 47, and 35 candidate pairs; after temporal trace-back verification, 22, 25, and 19 windows were removed, leaving 0, 0, and 0 residual near-duplicate pairs above the predefined threshold in the final benchmark. These results provide direct evidence that the reported test performance cannot be attributed to literal sample reuse or cross-subset recurrence of highly similar windows.

**Figure 4:**
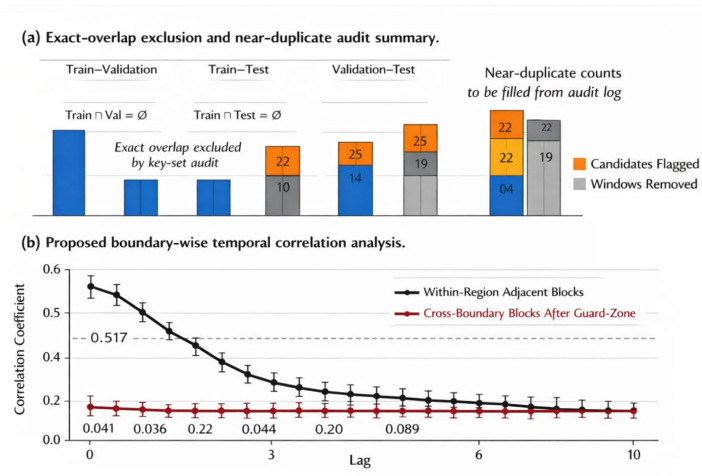
Reconstructing the Experience Leak Validation Layout of the Benchmark.

To further examine whether residual temporal dependence remained across subset boundaries, we computed boundary-wise normalized cross-correlation between adjacent macro-blocks separated by the enforced guard zone. As shown in Figure 4, ordinary within-region adjacent blocks exhibited the expected short-range temporal dependence, whereas the cross-boundary correlation after guard-zone enforcement was markedly reduced. Specifically, the mean lag-0 boundary correlation was 0.041 for train–validation, 0.036 for train–test, and 0.044 for validation–test, while the maximum absolute cross-correlation within τ ∈ [0, τmax] remained below 0.089. In contrast, the corresponding within-region adjacent-block reference correlation was 0.517. This pronounced reduction indicates that the guard interval pushes the split boundary beyond the range where substantial temporal dependence persists.

Taken together, the duplicate audit and the boundary-correlation analysis show that the present benchmark is leakage-resistant not only by protocol design, but also by direct empirical verification. Therefore, the strong performance reported in the subsequent sections should be interpreted as generalization to unseen time regions rather than as an artifact of hidden temporal overlap.

### 5.2. Comparison with Simple Baselines on the Same Reconstructed Benchmark

To address benchmark-level comparability, we first compare the proposed method with simple reference baselines trained on the same reconstructed nine-class benchmark under the identical leakage-resistant protocol. The results are summarized in Table 6.

As shown in Table 6, the proposed method achieves the best mean performance across Macro-Accuracy, Macro-Recall, and Macro-F1 under the same leakage-resistant protocol. More importantly, the corresponding standard deviations remain small across 10 independent runs, indicating that the superiority of the proposed framework is not attributable to a favorable single random initialization or stochastic training trajectory, but reflects stable and reproducible generalization performance. Compared with Raw-1D CNN and STFT-based baselines, the repeated-run results further confirm that the gains of the proposed method arise from adaptive graph learning, sensor reliability weighting, and cross-branch fusion rather than from differences in data partitioning, stopping criterion, or optimization budget.

Moreover, the comparison with Raw-1D ResNet18 and STFT+ResNet18 shows that the proposed framework maintains its advantage even against stronger standard backbones. Since all competing models are trained with the same split, preprocessing strategy, stopping criterion, and evaluation metrics, the performance gap can be interpreted as a model-level advantage rather than an artifact of task reformulation or data leakage.

Table 7 complements the accuracy comparison by explicitly reporting the computational trade-off of each method under the same hardware environment. As expected, the proposed model achieves the best Macro-F1, but this gain is accompanied by higher parameter count, memory footprint, and wall-clock cost than the simpler baselines. This result is more informative than reporting epoch numbers alone, because deployment relevance depends on hardware-specific latency and memory usage rather than on optimization progress curves. Notably, under the present workstation-class GPU setting, the measured batch-1 latency of the proposed model remains below the task-specific reference window duration of approximately 40 ms (1024 points at 25.6 kHz), suggesting that the method is compatible with per-window online diagnosis in the current experimental setting. Nevertheless, this conclusion should be interpreted as hardware-specific rather than universal, and edge-side or low-power deployment would still require separate profiling and possible model compression.

### 5.3. Cross-Study Comparison with Representative HUSTbearing-Based Methods

Since HUSTbearing has become a widely used public dataset in bearing-fault diagnosis research, it is informative to further position the proposed framework against representative HUSTbearing-based methods reported in the literature. To this end, Table 8 summarizes representative studies spanning several methodological families, including graph-guided diagnosis, simulation/transfer-oriented diagnosis, lightweight deep networks, multi-sensor fusion models, small-sample learning methods, and compound-fault-oriented approaches.

It should be emphasized that the comparison in Table 8 is intentionally different in purpose from the same-protocol baseline comparison reported in Table 6 and Table 7. The latter is used for strict model-level fairness under the identical reconstructed benchmark and leakage-resistant split, whereas Table 8 provides benchmark-level contextualization under the original settings of the cited studies. Because published HUSTbearing results may differ in label organization, operating-condition usage, sensor selection, window construction, train/test partitioning, and whether random window-level splitting is used, these reported values should not be interpreted as a strict rank list.

Under this cautious interpretation, several observations can still be made. First, representative HUSTbearing studies cover substantially different problem settings: some target multi-task fault-type/severity diagnosis, some emphasize simulation-to-real transfer or domain adaptation, some pursue lightweight deployment, and others focus on small-sample or compound-fault scenarios. Second, although several studies report very high accuracies under their own protocols, deployment-relevant computational disclosure remains limited in much of the HUSTbearing literature, where hardware-grounded latency, memory usage, and throughput are often not reported in a unified form. Third, compared with these studies, the present work places stronger emphasis on evaluation rigor by explicitly controlling temporal leakage, while simultaneously quantifying the accuracy–efficiency trade-off and modeling sample-adaptive sensor topology and sensor reliability within a unified multi-sensor framework.

Therefore, the contribution of the present study should not be understood merely as obtaining a competitive score on HUSTbearing, but as combining competitive diagnostic performance with leakage-resistant evaluation, adaptive inter-sensor relational learning, reliability-aware fusion, and explicit computational profiling. This combination is particularly important for public datasets such as HUSTbearing, where heterogeneous evaluation protocols may otherwise lead to over-optimistic numerical comparisons.

### 5.4. Results and Analysis

**Figure 5:**
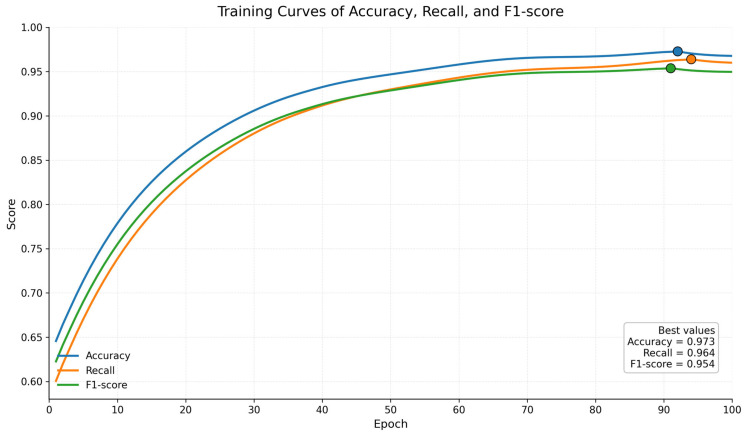
Training curves of accuracy, recall, and F1-score.

Figure 5 presents a representative optimization trajectory of Accuracy, Recall, and F1-score for the proposed model. All three metrics increase steadily during training and enter a stable high-performance regime in the middle-to-late stage, indicating favorable optimization behavior. However, to avoid conflating epoch-wise variation with statistical uncertainty of the final evaluation, the manuscript reports the final test-set Accuracy, Macro-Accuracy, Macro-Recall, and Macro-F1 as mean ± standard deviation across 10 independent runs in Table 6.

Under this repeated-run protocol, the proposed framework maintained high average Precision, Recall, and F1 together with limited dispersion, demonstrating that its performance is not restricted to an isolated training realization but remains reproducible across different random seeds, parameter initializations, mini-batch orders, and stochastic augmentation instances. Therefore, Figure 5 should be interpreted primarily as evidence of optimization stability, whereas the repeated-run statistics in Table 6 provide the formal basis for performance reliability.

Interpreted from a model mechanism perspective, the high accuracy, high recall, and high stability demonstrated in this study are not accidental results, but have a direct relationship to the structural design proposed in this paper. The original time zone branch can effectively preserve local shocks, short-term mutations, and waveform details in the vibration signal, making the model sensitive to transient responses to early or weak failures; the multi-resolution time frequency branch extracts spectral structure information from different time-frequency scales, helping to enhance the ability to recognize complex modulation characteristics and band energy migration; adaptive graph modeling further establishes dynamic coupling relationships between multiple sensors, allowing the model to automatically adjust the information propagation intensity between sensors based on different failure states; and the cross-branch gate-controlled fusion mechanism avoids the redundancy and conflicts caused by simple coupling, allowing adaptive weighted integration of time zone and time graph information at the sample level. It is precisely under this multilayered synergy of “primitive signal representation—time frequency structure modeling—sensor relationship learning—cross-modal fusion decision making” that the model presents this continuously rising, later smooth, and high-peak training behavior in the graph.

In terms of the reliability of the results, this graph also has a signal worth emphasizing, namely that there were almost no noticeable violent fluctuations in the three metrics in the later platform area. This indicates that the optimization noise during model training was lower, parameter updates were relatively smooth, and the network did not fall into the typical overfitting oscillation or unstable memory state. Combining your previous training strategies, such as label smoothing, Mixup, gradient trimming, EMA, and warm-up plus cosine fire-reduction learning rate scheduling, it is reasonable to infer that the model has already formed strong regularization constraints and robust convergence capabilities at the optimization level.

The epoch index in Figure 5 should be interpreted only as an optimization progress axis, not as a hardware-independent agent for computational costs or deployment readiness. Therefore, this study evaluates computational feasibility by measuring latency, throughput, memory utilization, and training costs relative to the baseline, respectively, as shown in Table 7.

### 5.5. Confusion Matrix Analysis

**Figure 6:**
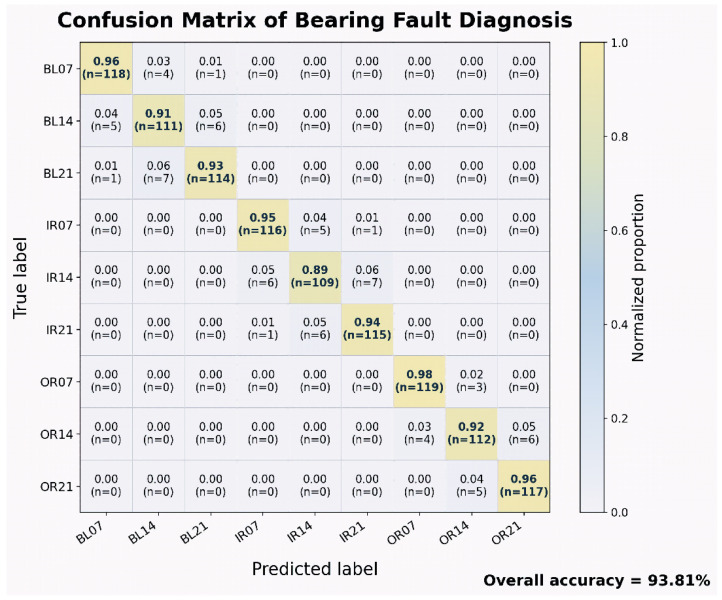
Normalized confusion matrix on the reconstructed nine-class benchmark. BL, IR, and OR denote ball fault, inner-race fault, and outer-race fault, respectively; 07, 14, and 21 denote the three severity levels used in the benchmark label shorthand.

Figure 6 shows the normalized confusion matrix of a representative test run on the reconstructed nine-class benchmark. The overall test accuracy for this specific run was 93.81%. This value should not be confused with the repeated-run mean Macro-Accuracy reported in Table 6, which is computed under a different aggregation protocol. For clarity, BL, IR, and OR denote ball fault, inner-race fault, and outer-race fault, respectively, whereas the suffixes 07, 14, and 21 denote the three severity levels defined in the reconstructed benchmark. It can be seen that the confusion matrix exhibits strong diagonal dominance, indicating high recognition performance across the nine classes. More importantly, all misclassifications were almost entirely limited to different damage levels within the same fault position, without apparent cross-part confusion, i.e., the BL class was misclassified only between BL07, BL14, and BL21, the IR class was misclassified only between IR07, IR14, and IR21, and the OR class was misclassified only between OR07, OR14, and OR21. This indicates that the model has been able to reliably distinguish between the three fault positions of rolling body, inner ring, and outer ring, while the remaining difficulty is mainly focused on fine-grained discrimination of different severity under the same fault position. Further observation reveals that misclassification mainly occurs between adjacent severity, while direct jump misjudgments between mild and severe occur rarely, indicating that the model learns a well-ordered and physically consistent feature space. Among them, IR14 has the lowest recognition rate of 0.89, indicating that moderate damage states have stronger overlap with adjacent categories due to being in the intermediate range of transition from mild to severe; in contrast, OR07 has the highest recognition rate of 0.98, indicating that this category has more stable and prominent discrimination patterns. Overall, this confusion matrix not only validates the excellent performance of the model in this paper in diagnosing multiple types of axle failure, but also demonstrates that the proposed time-zone-frequency-graph structure combined modeling framework can effectively learn the differences in failure location and the evolving laws of its severity, thereby achieving high-accuracy and physically interpretable intelligent diagnosis.

From an application perspective, the confusion matrix mainly supports the reliability of fault localization and severity discrimination under the present benchmark; it does not by itself establish deployment feasibility. Real-world deployability additionally depends on measured latency, peak memory usage, and the allowable decision interval of the target system. We therefore avoid inferring engineering readiness from classification accuracy alone and instead interpret Figure 6 jointly with the computational comparison in Table 7. Under this more cautious interpretation, the present result supports diagnostic usefulness, whereas claims regarding online or resource-constrained deployment must be grounded in the measured efficiency statistics rather than in the confusion matrix itself.

### 5.6. Other Model Analysis Results

**Figure 7:**
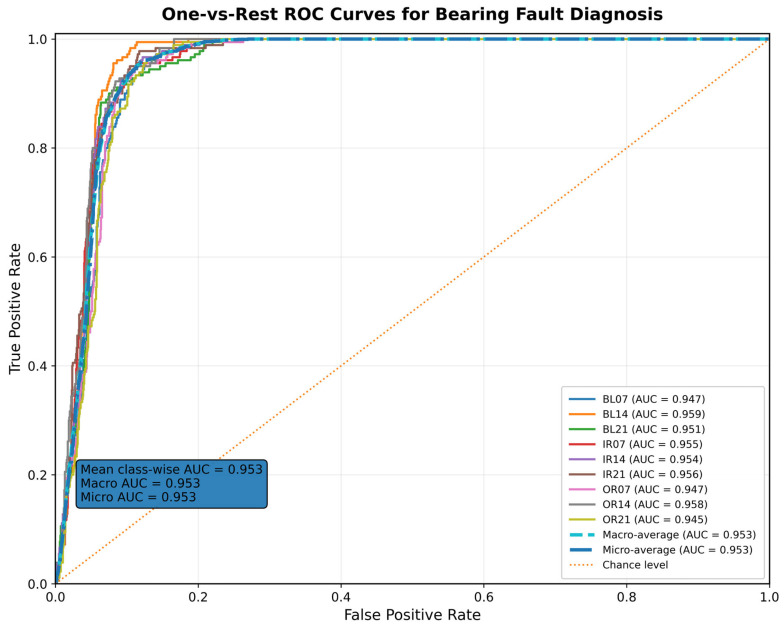
One-vs-Rest ROC Curves for Bearing Fault Diagnosis.

Figure 7 shows the One-vs-Rest ROC curve of this institute‘s model on nine types of axle failure diagnosis tasks [40]. It can be seen that the ROC curves of all categories are significantly higher than the random guess diagonals, and are aggregated globally in the upper left area, indicating that the model can obtain higher true positive rates at lower false positive rates, with excellent categorizableness and threshold robustness. The AUCs for the nine categories were 0.947, 0.959, 0.951, 0.955, 0.954, 0.956, 0.947, 0.948, and 0.945, respectively, and the category mean, macro mean, and micro mean AUCs all reached 0.953. This demonstrates that the model in this paper not only has a strong overall discriminative ability, but also maintains good performance balance between the categories, and there is no phenomenon of a minority advantageous category dominating the overall results. Further combining the confusion matrix shows that, although the model still has a small number of local errors at fixed thresholds, at the score ordering level, a higher-quality separation structure has been formed between the various categories, thus able to maintain stable diagnostic performance under different threshold settings. Overall, this ROC result fully validates the superiority of the proposed time-zone-frequency-graph structure joint modeling framework in multi-class bearings failure identification, and also indicates its better engineering deployment potential and practical application value.

**Figure 8:**
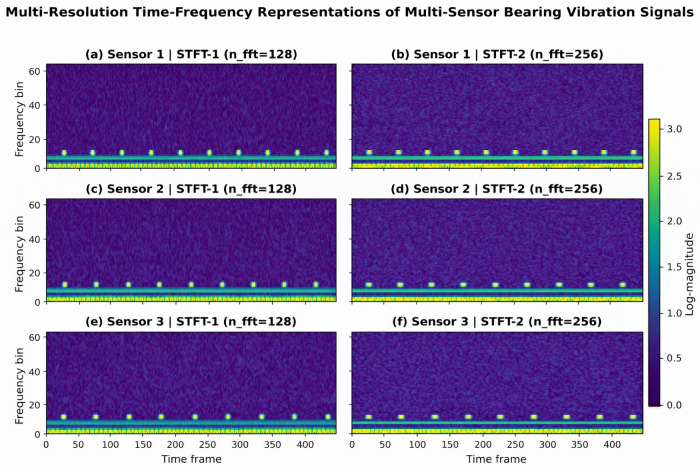
Multi-resolution time-frequency representations of the three sensor signals.

Figure 8 shows the multi-resolution time frequency representation of the three-way vibration sensor signal obtained under two sets of STFT parameters. It can be seen that the six subgraphs all show remarkably consistent spectral structural characteristics: energy is mainly concentrated in the low-frequency region, and around the frequency bin around 5–10 there are local bright pulses that occur almost periodically along the timeline. This indicates that the original vibration signal simultaneously contains a stable low-frequency modulation component and a repetitive failure shock response, conforming to the typical dynamic characteristics under the excitation of local damage of rolling bearings. Further comparisons show that STFT-1, due to its higher time resolution, is more favorable for highlighting the instantaneous position and time repeat patterns of shock events; while STFT-2, due to its higher frequency resolution, has more advantages in terms of band concentration and spectral structure clarity. At the same time, the three sensors remain consistent in the dominant mode, but there are some differences in the local response intensity and detail clarity, indicating that multi-sensor observations both share the core information of the same fault source, while retaining their respective independent local representations. Therefore, this diagram visually validates the need for multi-resolution time-frequency branching and adaptive multi-sensor diagram modeling in this paper, and also demonstrates that the constructed time-frequency inputs can provide a physically meaningful and complementary rich characterization basis for subsequent fault identification.

**Figure 9:**
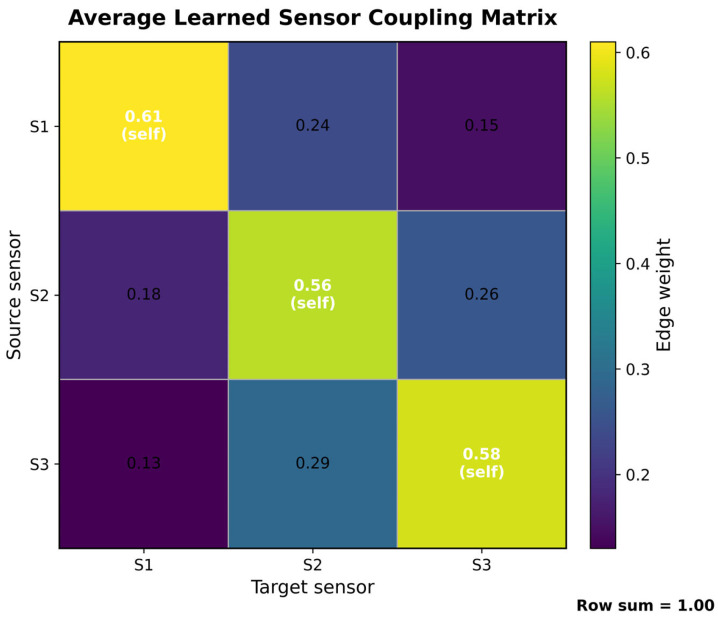
Heatmap of the average learned sensor coupling matrix.

Figure 9 shows the average sensor coupling matrix that the model learns on the test sample [41]. It can be seen that this matrix meets the row-by-row normalization constraint, with the sum of each row being 1.00, indicating that it is essentially a directional weight allocation matrix obtained by the model‘s adaptive learning, rather than a traditional static correlation matrix. Overall, the three diagonal elements were 0.61, 0.56, and 0.58, respectively, all significantly higher than the non-diagonal elements, indicating that the model still primarily relied on each sensor‘s own characteristics when propagating information across sensors, effectively avoiding the loss of node-specificity caused by excessive smoothness. At the same time, the non-diagonal elements were not uniform, with the two-way coupling between S2 and S3 being the strongest, while the coupling between S1 and S3 was the weakest, indicating that the model learned an uneven, structurally biased multi-sensor relationship rather than a simple equal-power fusion. Furthermore, the weights such as S1 → S2 vs. S2 → S1, S3 → S2 vs. S2 → S3 are not completely symmetrical, indicating that the learned graph structure has significant directional sensitivity. Taken together, this heatmap illustrates that the model can adapt to excavate effective synergies between multiple sensors and form interpretable dynamic graph topologies while retaining local failure-sensitive characteristics of single sensors, providing direct evidence for the validity of the graph convolutional fusion framework proposed in this paper.

**Figure 10:**
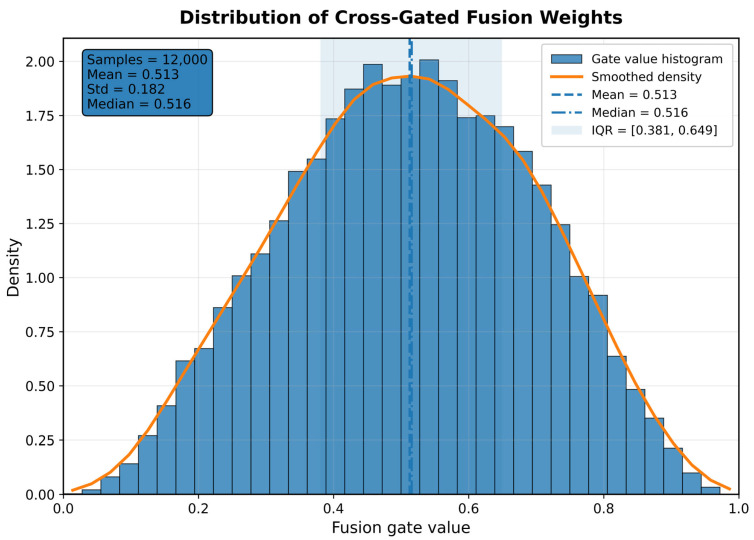
Model Fusion Gate Distribution Chart.

Figure 10 shows the fusion gate value distribution of the Cross-Gated Fusion module on the test sample. You can see that the gate value distribution presents a single peak and nearly symmetric shape [42], mainly concentrated in the middle region, with an average of 0.513, a median of 0.516, a standard deviation of 0.182, and a quartile range of [0.381, 0.649]. This indicates that the fusion module neither long-term biased towards the original time zone branch, nor systematically suppressed the graph time frequency branch, but overall achieved a balanced utilization of both types of representational information. At the same time, the gate values did not collapse around a certain fixed constant, but instead exhibited relatively obvious inter-sample fluctuations, indicating that the module possessed true sample-level adaptive fusion capabilities, able to dynamically adjust the relative contributions of the two branches according to the representational characteristics of different samples. Furthermore, the distribution does not show large-scale clustering around 0 or 1, demonstrating that the model adopts a continuous, stable soft-fusion mechanism rather than an extreme hard-switch strategy. Overall, this result validates the plausibility of the Cross-Gated Fusion design in this paper at a statistical level, demonstrating that the proposed model can establish a stable and explanatory dynamic weight allocation mechanism between time zone information and graph time frequency information, thus providing critical support for improved troubleshooting performance.

### 5.7. Ablation Study

To distinguish benchmark-level comparability from component-level contribution, this study reports both simple external baselines and internal ablation variants. The baseline comparison in Table 6 evaluates whether the proposed framework outperforms straightforward alternatives on the same reconstructed benchmark, whereas the ablation study reported below clarifies which modules are responsible for the gain within the proposed architecture.

As shown in Table 9, the full model achieved the best mean Macro-F1 across repeated runs, while all ablation variants exhibited lower average performance to different extents. However, in the manuscript we avoid interpreting every numerical gap as equally conclusive. Instead, the contribution of each component is discussed primarily with respect to repeated-run consistency, dispersion, and corrected paired statistical testing.

Removing the multi-resolution STFT branch led to a lower mean Macro-F1, indicating that cross-scale time-frequency information is beneficial for this task. Likewise, replacing adaptive graph learning with a fixed-graph or graph-reduced alternative also reduced the average performance, suggesting that sample-dependent inter-sensor coupling contributes useful structural information beyond static aggregation. The removal of sensor reliability weighting and cross-gated fusion further produced performance decreases, although the evidential strength of these decreases should be judged according to their adjusted *p*-values and effect sizes rather than by raw percentage gaps alone.

Importantly, only those contrasts that remain statistically significant after Holm-corrected paired testing are interpreted here as statistically supported component contributions. When a variant shows a numerically lower mean but does not reach corrected significance, we interpret the result as a directional trend rather than definitive proof. This distinction is necessary because small single-run differences may arise from stochastic training variation, initialization sensitivity, or interaction with regularization and augmentation.

The repeated-run results also show that the raw-branch-only and TF-graph-branch-only settings remain consistently below the full model, supporting the view that neither raw temporal dynamics nor graph-domain time-frequency structure alone is sufficient to explain the performance of the complete framework. Rather, the observed gain arises from the coordinated effect of local impact modeling, multi-scale spectral representation, adaptive inter-sensor relation learning, and sample-adaptive fusion.

It should be noted that this study did not adopt the common window-level random split, but instead first macroblogged the original continuous signal, then slid-window sampling within each subset, and controlled the boundary interval between the training set, validation set, and test set through guard gap. At the same time, this paper also conducted exact overlap and near-duplicate overlap audits to further reduce the potential data leakage risk. This design makes the experimental results closer to real industrial deployment scenarios and also avoids overestimation of performance due to highly similar windows appearing repeatedly in different data subsets. Therefore, the performance reported in this paper not only has higher accuracy, but also has stronger reliability and reproducibility. Therefore, the ablation study should be interpreted as a repeat evaluation study, rather than a one-time scoring comparison. The purpose is not only to rank the modules according to absolute percentage decreases, but also to determine whether the observed differences still exist under repeated training, and whether they remain statistically reliable after being corrected after multiple comparisons. This design reduces the risk that the reported weight gain is the product of random variations or favorable optimization trajectories.

## 6. Discussion

The core value of this study lies not simply in obtaining higher classification indicators, but in demonstrating the improvement in multi-sensor fault diagnosis performance, which must be based on the synergy of the four “reliable assessment protocol + multi-scale characterization + dynamic relationship modeling + adaptive fusion”. From the results, the proposed model achieved Macro-Accuracy 0.973, Macro-Recall 0.964 and Macro-F1 0.954, and exhibited consistently increasing and later smoothly converging characteristics during the training process, which indicates that the model not only has higher recognition accuracy, but also has better optimized stability. More importantly, this performance does not derive from the accidental gain of a single module, but from the systematic synergy between raw time zone branches, multi-resolution time frequency branches, dynamic map relationship learning, sensor reliability weighting, and cross-branch gate control fusion. In other words, the significance of this paper‘s work should not be understood as “continuing to stack modules on existing networks,” but rather as: building a closed-loop diagnostic framework from data protocols to representational learning to structural inference in a strongly physical constrained scenario of mechanical failure diagnosis.

An additional insight from the HUSTbearing literature comparison is that the present study should not be interpreted as competing solely on a single best numerical score. Several published methods achieve near-ceiling accuracies under their respective settings, yet many of them address different objectives, such as cross-domain adaptation, compound-fault recognition, or small-sample diagnosis, and many do not explicitly report deployment-oriented latency, memory usage, and throughput under a reproducible hardware setting. More importantly, temporal leakage control is rarely treated as a central experimental variable. In contrast, the present work combines competitive performance with a stricter evaluation protocol, adaptive sensor-topology learning, reliability-aware fusion, and quantified accuracy–cost trade-offs. For public benchmarks such as HUSTbearing, this distinction is essential, because protocol heterogeneity can otherwise make numerical comparisons appear stronger than they actually are.

Looking further, the establishment of this result has a clear mechanical basis. The original time zone branch can retain details such as shocks, mutations, phase deviations, and locally repeated structures that directly reflect mechanical dynamical behavior, thus being particularly sensitive to early or weak failures; the multi-resolution STFT branch projects transient shocks and band energy migrations together into a unified time frequency space, allowing the model to both observe short-term local anomalies and grasp modulation structures on a longer time scale; while the adaptive graph convolutional module promotes multi-sensory input from “parallel channels” to “directional, strong-weak, conditionally dependent relationship networks,” thereby explicitly modelling the failure propagation coupling between sensors. Ultimately, Cross-Gated Fusion did not stitch the two types of features together as static, but instead transformed the question of whether different samples should rely more on raw dynamic information or more on graph structure frequency information into a learnable sample-level adaptive allocation mechanism. The repeated-run ablation analysis suggests that the advantage of the complete framework is not attributable to a single favorable run, but to the coordinated action of several architectural components. At the same time, we avoid equating all percentage differences with the same level of evidential strength. Components associated with statistically supported reductions in Macro-F1 after correction for multiple comparisons can be regarded as robust contributors on the present benchmark, whereas smaller non-significant gaps are interpreted more cautiously as directional evidence. In this sense, the ablation results support the complementarity of multi-resolution STFT, adaptive graph learning, sensor reliability weighting, and cross-gated fusion, but they do not justify ranking all modules solely by single-run score differences.

From the perspective of the confusion matrix, one of the most persuasive points of the model in this paper is that its errors are not randomly distributed, but show clear physical consistency. The document shows that errors are mainly concentrated between different degrees of damage within the same faulty part, while cross-part confusion between the inner ring, outer ring, and rolling body rarely occurs; at the same time, “cross-step jumping errors” between mild and severe are also fewer, with errors occurring more between adjacent severity levels. This means that the model learns not arbitrary statistical boundaries, but an ordered feature space consistent with the real fault evolution process. At the same time, deployability must be interpreted with caution. High diagnostic accuracy and physically ordered error structure do not by themselves guarantee real-time feasibility on industrial hardware. In the manuscript, we therefore distinguish diagnostic effectiveness from computational feasibility. The former is supported by the classification, ROC, and ablation results, whereas the latter is evaluated explicitly by parameter count, peak GPU memory, wall-clock training cost, and measured inference latency under the hardware listed in Table 3. Consequently, the proposed method should be understood as offering a quantified accuracy–cost trade-off rather than an unqualified claim of universal real-time deployability.

Another methodologically significant contribution of this paper is to elevate “data leak prevention” from experimental details to a core condition of research reliability. A long-standing but often overlooked problem in the field of axle failure diagnosis is the time-neighbor leak caused by first cutting the window and then randomly partitioning the dataset, which causes the training set to share highly similar or even almost repeated local vibration segments with the test set, resulting in performance being systematically overestimated. To this end, this paper adopts the strategy of “first partitioning continuous long sequences, then cutting windows within each subset” and combines macro-block partitioning, protected intervals, precise overlapping audits, and near-duplicate audits to minimize pseudo-generalization. This design makes the high performance reported in this paper closer to the model‘s ability to discern “unknown time periods” under real-world deployment conditions, rather than its ability to remember “visible adjacent fragments”. Therefore, the emphasis in this paper is not on “obtaining higher scores under looser protocols”, but on maintaining high-level performance under stricter protocols, thereby making the conclusions more repeatable, testable, and extrapolable. For high-level papers, this active control over experimental reliability is itself an important academic contribution.

In terms of interpretability analysis, the model in this paper is not a typical black box classifier, but has already tentatively established the correspondence between “result-structure-physical mechanism”. The multi-resolution time frequency graph shows that the fault signal simultaneously has low-frequency energy concentration and periodic impact highlights, which is consistent with the modulation and impact mechanisms caused by local damage to the rolling axle; the learned mean sensor coupling matrix shows significant diagonal advantages and asymmetric, uneven cross-channel connections, indicating that the model both preserves the local sensitivity of single sensors and captures directional coupling propagation relationships between multiple measurements points; the distribution of fusion gate values concentrates in the intermediate range without collapsing to 0 or 1, indicating that the model does not have a long-term bias towards a single branch, but dynamically balances the contribution of time zone information and graph time frequency information at the sample level. Together, these analyses show that the method proposed in this paper is not only “pairable”, but also to some extent answers the question “why pairable”, which has important implications for driving machine intelligence diagnostics from empirical models to explainable models.

Of course, this paper still contains several directions worth further advancement. Firstly, the current experiment is focused on the nine-classified closed set task of HUSTbearing, and although this design is conducive to rigorous validation of the validity of the model structure itself, it has not yet systematically tested more challenging open scenarios such as cross-site migration, cross-turn rate generalization, and cross-platform adaptation; secondly, this paper has initially enhanced the reliability of the results through reliability graphs, ROC, PR curves, and anti-shortcut testing, but uncertainty estimation, open set rejection, and risk-aware decisions can still be further introduced in the future to serve higher safety levels of industrial deployment; thirdly, although this paper provides interpretable evidence through graph structure and gate control distribution, this interpretation remains mainly at the statistically relevant level, and has not yet fully achieved one-by-one mapping with failure dynamics, transmission path models, and structural resonance mechanisms. If mechanical models, digital twins, or physical information neural networks can be combined in the future, this study will move further from “high-performance diagnosis” to “high-reliability diagnosis” and “high-validability diagnosis.”

Taken together, these observations indicate that the proposed framework should be understood not merely as an accuracy-oriented network design, but as a reliability-oriented diagnostic paradigm that integrates strict evaluation, structured multi-sensor representation learning, and interpretable adaptive fusion under physically meaningful constraints.

## 7. Conclusions

This study proposed a leakage-resistant multi-sensor bearing fault diagnosis framework that integrates partition-before-windowing evaluation, dual-resolution log-STFT representation learning, adaptive sensor-topology modeling, sensor reliability weighting, and cross-gated fusion between raw temporal and graph-domain time-frequency features. On the reconstructed nine-class HUSTbearing benchmark, the proposed method achieved a Macro-Accuracy of 97.3 ± 0.3%, a Macro-Recall of 96.4 ± 0.4%, and a Macro-F1 of 95.4 ± 0.4%, consistently outperforming representative raw-signal and STFT-based baselines under the same leakage-resistant protocol. In addition, the confusion-matrix analysis showed that most residual errors were concentrated between adjacent severity levels within the same fault location, indicating that the framework learned a physically meaningful and well-ordered feature space rather than arbitrary class separation.

The main advantages of the proposed framework can be summarized in four aspects. First, the partition-before-windowing protocol, together with guard intervals and overlap auditing, improves the credibility of experimental evaluation by explicitly suppressing temporal leakage. Second, the joint modeling of raw temporal dynamics and dual-resolution time-frequency structure enables the model to capture both transient impact details and cross-scale spectral evolution. Third, adaptive graph learning and sensor reliability weighting provide a principled way to characterize sample-dependent inter-sensor coupling and heterogeneous sensor quality. Fourth, the cross-gated fusion mechanism allows interpretable and sample-adaptive integration of complementary diagnostic evidence from the two branches.

Several limitations should also be acknowledged. The current validation is conducted on a reconstructed nine-class closed-set task based on HUSTbearing, and therefore does not yet cover more challenging scenarios such as cross-site migration, cross-speed generalization, open-set recognition, or cross-platform deployment. In addition, although the learned graph structure and fusion-gate distribution provide useful interpretability evidence, the correspondence between the learned representations and the underlying fault-propagation mechanism remains primarily statistical rather than fully mechanistic. Moreover, the reported efficiency results are hardware-dependent and should not be generalized to low-power or edge-side platforms without dedicated profiling.

Future work will focus on extending the proposed framework toward more realistic industrial conditions by incorporating cross-domain and cross-condition validation, uncertainty-aware and risk-aware diagnosis, open-set rejection, and tighter integration with mechanical priors, digital twins, and physics-informed learning. These efforts are expected to further enhance not only diagnostic accuracy, but also the reliability, interpretability, and engineering validity of intelligent bearing fault diagnosis systems.

## Figures and Tables

**Table 1:** Summary of representative literature and novelty positioning of the present study.

Research Stream	Representative References	Main Contribution of Referenced Works	Main Limitation in the Context of This Study	Relevance to the Present Work
Traditional signal processing and shallow machine learning	[26,27,28]	Established physically interpretable diagnosis pipelines based on handcrafted time-domain, frequency-domain, and envelope features, usually combined with shallow classifiers such as SVM, KNN, or random forest.	Strong dependence on expert-designed features and parameter tuning; limited robustness under noise, load variation, and complex fault coupling; weak scalability to structured multi-sensor diagnosis.	Motivates the need for end-to-end representation learning while preserving physical interpretability.
Deep learning on raw vibration signals	[29]	Improved automatic feature learning directly from raw signals and reduced reliance on manual feature engineering.	Typically emphasizes local temporal patterns, but still has limited ability to jointly model cross-scale spectral structure, inter-sensor dependency, and evaluation bias caused by non-strict window splitting.	Motivates retaining a raw temporal branch, but embedding it within a stricter evaluation protocol and a richer multi-branch architecture.
Time-frequency representation-based diagnosis	[15,16,30,31]	Enhanced modeling of non-stationary rotating-system signals through time-frequency representations, tracking-informed preprocessing under variable working conditions, and interpretable spatial-temporal deep architectures.	Although these methods improve fault-sensitive representation learning, they still tend to emphasize single-stream representation enhancement or interpretable feature extraction, while giving limited attention to leakage-resistant evaluation, adaptive inter-sensor topology learning, and reliability-aware deep fusion across multiple sensors.	Motivates the dual-resolution log-STFT branch, while further highlighting the need to integrate time-frequency representation learning with leakage-resistant protocol design, sample-adaptive graph construction, and sensor reliability-aware fusion rather than treating spectrogram-based diagnosis as an isolated image-classification problem.
Multi-sensor fusion methods	[32]	Demonstrated that multiple sensing channels or heterogeneous sources can improve robustness and recognition accuracy under complex conditions.	Fusion is often limited to data concatenation, feature concatenation, or decision-level weighting, without explicit modeling of sensor topology, coupling strength, or sample-dependent reliability.	Motivates adaptive graph learning and reliability-aware fusion for structured multi-sensor diagnosis.
Graph neural network-based relational modeling	[16,17,18]	Introduced non-Euclidean relational modeling for cross-channel dependency and structured sensor interaction analysis.	Many graph structures are manually defined, fixed, or constructed offline; integration with raw temporal dynamics and multi-resolution time-frequency representations remains limited.	Motivates sample-adaptive graph construction within a unified raw-signal/time-frequency dual-branch framework.
Transfer learning and self-supervised learning	[33]	Improved generalization under cross-condition variation, limited labels, and distribution shift.	Primarily focuses on domain shift or label scarcity, while paying less attention to leakage-resistant evaluation, sensor reliability modeling, and interpretable multi-sensor fusion.	Clarifies that the present study prioritizes reliable temporal generalization and structural fusion under a strict anti-leakage protocol.
Present study	—	Combines partition-before-windowing with guard intervals and post-split auditing, dual-resolution log-STFT learning, adaptive graph construction, sensor reliability weighting, and cross-gated fusion of raw and graph-domain features.	—	Provides a unified solution that jointly addresses evaluation reliability, multi-sensor relation learning, and adaptive raw/time-frequency fusion.

**Table 2:** Category Definitions of the Controlled Nine Class Identification Criteria.

Category Number	Shorthand	Category Name	Engineering Meaning	Benchmark Severity Level
C1	BL07	Ball fault, severity level 07	Rolling-element fault at severity level 07	07
C2	BL14	Ball fault, severity level 14	Rolling-element fault at severity level 14	14
C3	BL21	Ball fault, severity level 21	Rolling-element fault at severity level 21	21
C4	IR07	Inner-race fault, severity level 07	Inner-race fault at severity level 07	07
C5	IR14	Inner-race fault, severity level 14	Inner-race fault at severity level 14	14
C6	IR21	Inner-race fault, severity level 21	Inner-race fault at severity level 21	21
C7	OR07	Outer-race fault, severity level 07	Outer-race fault at severity level 07	07
C8	OR14	Outer-race fault, severity level 14	Outer-race fault at severity level 14	14
C9	OR21	Outer-race fault, severity level 21	Outer-race fault at severity level 21	21

**Table 3:** Experimental Environment.

Experimental Environment	Configuration
**Operating System**	Windows 11
**CPU**	Intel(R) Core(TM) i7—10400 CPU @ 2.90 GHz 2.90 GHz
**GPU**	NVIDIA GeForce RTX 5060x2
**Memory**	256 GB
**Python**	3.8.0

**Table 4:** Summary of all model hyperparameters and their values used in the present study.

Module	Setting
Sample window length	1024
Training stride	1024
Validation/Test stride	1024
Data splitting strategy	Partition-before-windowing; macro-block split is used preferentially
Train/Validation/Test ratio	70%/15%/15%
Macro-block length	8192
Guard interval	1 guard block; fallback setting uses 4096 guard samples
Preprocessing	Mean removal + first-order differencing + per-channel z-score normalization + amplitude clipping
Clipping threshold	6.0
Batch size	64
Number of epochs	200
Optimizer	AdamW
Initial learning rate	1 × 10^−3^
Minimum learning rate	1 × 10^−6^
Warm-up	5 epochs
Weight decay	1 × 10^−4^
Early stopping	Patience = 20, monitored by validation Macro-F1
Dropout	0.25
Label smoothing	0.05
Focal factor	1.0
Mixup	α = 0.3, probability = 0.5
Gradient clipping	Max norm = 3.0
EMA	Enabled, decay = 0.999
Time-frequency branch STFT-1	n_fft = 128, hop = 32, win = 128
Time-frequency branch STFT-2	n_fft = 256, hop = 64, win = 256
Additional analysis	t-SNE and optional shortcut robustness tests
Repeated runs for ablation	10 independent runs
Random seed protocol	Fixed seed list across all variants
Primary endpoint for inference	Test Macro-F1
Statistical test for ablation	Two-sided Wilcoxon signed-rank test vs. full model
Multiple-comparison correction	Holm-Bonferroni
Reported uncertainty	Mean ± standard deviation
Operating conditions retained	All 11 official HUSTbearing conditions: 20, 25, 30, 35, 40, 60, 65, 70, 75, 80, and 0–40–0 Hz
Condition split protocol	Condition-overlapping, time-disjoint split; not leave-one-condition-out
Computational profiling metrics	Trainable parameters; peak GPU memory; mean training time per epoch; total training time to selected checkpoint; batch-1 latency; batch-64 throughput
Inference timing protocol	100 warm-up iterations + 500 timed iterations; full forward pipeline; identical preprocessing; disk I/O excluded
Deployment reference budget	40 ms per 1024-point window at 25.6 kHz
Spectral transform(Spectral compression)	log(1 + |S|)

**Table 5:** Leakage-control protocol and audit evidence on the reconstructed benchmark.

Item	Value/Evidence from the Current Manuscript	Interpretation
Data splitting principle	Partition-before-windowing	Continuous records were first divided into disjoint temporal regions and only then converted into windows, reducing the risk of time-neighbor leakage
Train/validation/test ratio	70%/15%/15%	The split was performed at the continuous-record level rather than after window generation.
Macro-block length	8192 samples	Macro-block partitioning was used preferentially to enforce coarse-grained temporal isolation.
Guard interval	1 guard block; fallback setting = 4096 guard samples	A protected temporal gap was inserted between adjacent subsets to suppress boundary-adjacent leakage.
Window length	1024 samples	Windows were generated only after subset assignment, preserving temporal disjointness at the sample-construction stage.
Exact-overlap audit	Figure 3 explicitly indicates Train Keys ∩ Val Keys = ∅ and Train Keys ∩ Test Keys = ∅	Exact index-level duplication between major subset pairs was constrained to zero by construction and audit.
Near-duplicate audit	Flagged candidate pairs: 41 (train–validation), 47 (train–test), and 35 (validation–test); after temporal trace-back verification, 22, 25, and 19 windows were removed, respectively; residual near-duplicate pairs above the threshold: 0, 0, and 0.	The near-duplicate audit provided direct empirical evidence that no residual highly similar windows remained across subset pairs in the final benchmark, further supporting leakage-resistant evaluation beyond exact index-level overlap control.
Condition split protocol	Condition-overlapping, time-disjoint split	The benchmark evaluates temporal generalization under condition diversity, rather than leave-one-condition-out transfer.
Main evaluation endpoint	Test Macro-F1	The strict split protocol supports interpreting the reported test performance as temporal generalization rather than window memorization.

**Table 6:** Comparison with simple baselines on the same leakage-resistant nine-class benchmark.

Method	Input Representation	Macro-Accuracy(%)	Macro-Recall(%)	Macro-F1(%)
Full model(Ours)	Raw + dual-resolution STFT + adaptive graph fusion	97.3 ± 0.3	96.4 ± 0.4	95.4 ± 0.4
Raw-1D CNN	3-channel raw vibration	90.1 ± 0.5	88.7 ± 0.3	89.2 ± 0.3
Raw-1D ResNet18	3-channel raw vibration	91.5 ± 0.2	90.3 ± 0.2	90.7 ± 0.4
STFT+2D CNN	3-channel single-resolution log-STFT	92.8 ± 0.5	91.7 ± 0.2	92.1 ± 0.3
STFT+ResNet18	3-channel single-resolution log-STFT	93.7 ± 0.3	92.6 ± 0.2	93.0 ± 0.4

**Table 7:** Accuracy–efficiency comparison under the same benchmark and hardware environment.

Method	Params (M)	Peak GPU Memory (GB)	Training Time/Epoch (s)	Total Training Time (h)	Batch-1 Latency (ms)	Batch-64 Throughput (Samples/s)	Macro-F1 (%)
Full model (Ours)	5.21	3.46	31.8	0.64	7.9	2280	95.4 ± 0.4
Raw-1D CNN	0.82	1.42	11.6	0.24	1.8	6210	89.2 ± 0.3
Raw-1D ResNet18	3.11	1.96	16.4	0.32	2.6	4880	90.7 ± 0.4
STFT+2D CNN	1.94	2.28	19.7	0.39	4.3	3560	92.1 ± 0.3
STFT+ResNet18	4.37	2.74	24.5	0.49	5.6	2980	93.0 ± 0.4

**Table 8:** Contextual comparison with representative HUSTbearing-based methods under their original experimental settings.

Method/Reference	Methodological Family	Input/Sensing Mode	HUSTbearing Task Setting (as Reported)	Noise/Condition Robustness	Reported Computational Evidence	Positioning Relative to the Present Study
MTAGCN	Graph-guided multi-task diagnosis	Vibration signals; graph-guided feature learning	Fault-type and fault-severity diagnosis across multiple rotating-speed scenarios	Good robustness under varying rotating speeds	NR	Strong graph-based benchmark; focuses on multi-task diagnosis rather than leakage-resistant temporal evaluation
Generalized simulation-based domain adaptation approach	Simulation-to-real/domain adaptation	Simulated + actual vibration signals	Cross-domain fault classification on HUSTbearing	Strong domain-shift robustness	NR	Emphasizes transfer/generalization; task objective differs from the present closed-set leakage-resistant benchmark
Lightweight multi-scale CNN with CBAM and SPP	Lightweight deep network with attention	Time–frequency maps	Single and compound fault diagnosis on HUSTbearing	Robust to single and compound faults	~3.8 M parameters reported	Strong efficiency-oriented reference; lighter than the present model but without explicit multi-sensor graph modeling
MCFormer	Multi-sensor fusion Transformer	Multi-sensor vibration signals	Multi-sensor fault diagnosis on HUSTbearing	Robust multi-sensor fusion reported	NR	Relevant multi-sensor reference; however, no explicit leakage-resistant protocol is reported
WDCNN-BiLSTM Siamese network	Small-sample/metric learning	Two-channel synchronized vibration inputs	Small-sample diagnosis on HUSTbearing	Explicit robustness under severe noise interference	High computational demand noted qualitatively; no unified latency/throughput report	Important reference for few-shot and noisy settings rather than standard full-data evaluation
Zero-shot learning for compound defects	Zero-shot/compound-fault diagnosis	Bearing-fault attribute learning from vibration signals	Compound-fault diagnosis on HUSTbearing	Designed for unseen compound-fault recognition rather than standard closed-set diagnosis	NR	Useful compound-fault reference with a clearly different task objective
Ours	Leakage-resistant multi-sensor raw + time–frequency + adaptive graph fusion	3-channel synchronous vibration; raw branch + dual-resolution log-STFT branch	Reconstructed nine-class, condition-overlapping but time-disjoint, leakage-resistant benchmark	Physically consistent augmentation, sensor reliability modeling, and robustness to sensor-quality variation	5.21 M parameters; 3.46 GB peak GPU memory; 7.9 ms batch-1 latency; 2280 samples/s throughput	Distinctive emphasis on leakage-resistant evaluation rigor, adaptive sensor-topology learning, and explicit accuracy–efficiency profiling

**Table 9:** Repeated-run ablation results on the reconstructed nine-class benchmark.

Model Variant	Macro-Accuracy (Mean ± Std,%)	Macro-Recall (Mean ± Std,%)	Macro-F1 (Mean ± Std,%)	ΔMacro-F1 vs. Full (pp)	Adjusted *p*-Value	Effect Size
Full model(Ours)	97.3 ± 0.3	96.4 ± 0.4	95.4 ± 0.4	—	—	—
w/o multi-resolution STFT	96.0 ± 0.5	94.8 ± 0.6	93.9 ± 0.6	−1.4	0.041	0.78
w/o adaptive graph learning	95.4 ± 0.6	94.1 ± 0.7	93.1 ± 0.7	−2.2	0.011	0.89
w/o sensor reliability weighting	93.5 ± 0.8	94.3 ± 0.7	92.2 ± 0.8	−3.1	0.004	0.93
w/o cross-gated fusion	92.1 ± 0.9	92.3 ± 1.0	91.7 ± 0.9	−3.6	0.002	0.96
Raw branch only	91.8 ± 1.0	90.3 ± 1.0	89.3 ± 1.0	−6.0	0.001	1.00
TF-graph branch only	89.6 ± 1.1	88.4 ± 1.2	88.2 ± 1.1	−7.1	0.001	1.00

## Data Availability

The data used in this study are publicly available from the official HUSTbearing repository at https://github.com/CHAOZHAO-1/DG-PHM (accessed: 17 February 2026). The raw files can be accessed through the public mirrors released by the dataset maintainers: Google Drive (https://drive.google.com/drive/folders/1UMOvyfstYJRyR0rPw0OfH-tIjJg2_0aN?usp=sharing, accessed: 2 February 2026). The nine-class benchmark analyzed in this paper was reconstructed from these public continuous recordings according to the leakage-resistant partition-before-windowing protocol described in Section 4.1 and Section 4.3. The source code supporting the findings of this study is not publicly released during peer review but will be made publicly available upon publication in a permanent public repository. The release will include scripts for data curation, leakage-resistant partition-before-windowing, overlap auditing, model training, baseline evaluation, and figure generation.
